# Do health professionals value genomic testing? A discrete choice experiment in inherited cardiovascular disease

**DOI:** 10.1038/s41431-019-0452-z

**Published:** 2019-06-11

**Authors:** James Buchanan, Edward Blair, Kate L. Thomson, Elizabeth Ormondroyd, Hugh Watkins, Jenny C. Taylor, Sarah Wordsworth

**Affiliations:** 1 Health Economics Research Centre, Nuffield Department of Population Health, University of Oxford, Old Road Campus, Oxford, OX3 7LF UK; 2 National Institute for Health Research Biomedical Research Centre, Oxford, UK; 3 Oxford Centre for Genomic Medicine, Oxford University Hospitals NHS Foundation Trust, Oxford, UK; 4 Department of Clinical Genetics, Churchill Hospital, Headington, Oxford UK; 5 Oxford Medical Genetics Laboratory, Oxford University Hospitals NHS Foundation Trust, The Churchill Hospital, Oxford, UK; 6 Radcliffe Department of Medicine, University of Oxford, Oxford, UK; 7 Division of Cardiovascular Medicine, Radcliffe Department of Medicine, University of Oxford, Oxford, UK; 8 Wellcome Trust Centre for Human Genetics, University of Oxford, Oxford, UK

**Keywords:** Economics, Next-generation sequencing, Cardiovascular diseases, Genomics, Health care economics

## Abstract

Next generation sequencing (NGS) approaches are moving from research into clinical practice. However, the optimal NGS approach in well-defined adult-onset familial diseases, such as inherited cardiovascular disease, remains unclear. We aimed to determine which attributes encouraged or discouraged the uptake of genomic tests in this context, and whether this differed by test type. We conducted a web-based discrete choice experiment in health professionals in the UK who order NGS tests for inherited cardiovascular disease. Respondents completed 12 hypothetical choice tasks in which they selected a preferred test from four alternatives: whole genome sequencing, whole exome sequencing, panel testing and genetic testing not indicated. Tests were specified in terms of five attributes: diagnostic yield, detection rate for variants of unknown significance, cost, quantity of counselling received and disclosure of secondary findings. Mixed logit regression analysis was used to analyse the choice data. We found that uptake of NGS increases if tests identify more pathogenic mutations, identify fewer variants of unknown significance, or cost less. Respondents were willing to pay £117 for every 1% increase in diagnostic yield. Considerable heterogeneity was observed around preferences for several test attributes. Overall, panel testing had the highest predicted uptake rate. Our results indicate that NGS tests are valued by health professionals for well-defined adult-onset familial diseases, however, these professionals have strong preferences for panel testing rather than whole genome sequencing and whole exome sequencing. This finding suggests that different uptake rates should be explicitly modelled when designing and evaluating future genomic testing services.

## Introduction

The first human genome sequence was produced in 2003, but the cost of sequencing an entire genome remained prohibitively high for routine clinical use until the development of next generation sequencing (NGS) approaches in 2008. These approaches allow either the whole genome (via whole genome sequencing (WGS) or parts of it (via whole exome sequencing (WES) or targeted panels) to be sequenced faster, at great depth and increasing sensitivity [1, 2]. Given that the costs of WGS, WES and gene panel testing have continued to fall over time, attention is now starting to turn towards the translation of these genomic tests into clinical practice.

A key step in the translation process is to understand the views and preferences of key stakeholders with respect to genomic testing, including both patients and health professionals. This information can help decision-makers to predict the likely uptake of genomic testing in different clinical contexts, which will impact on the cost effectiveness of such tests. The preferences of health professionals are particularly important in the context of genomic testing because in many countries patient access to these tests is strictly controlled by specialist healthcare teams.

The use of genetic data to guide diagnosis, prognosis and treatment decisions is routine practice in inherited cardiovascular diseases such as cardiomyopathy and cardiac ion channel disorders. These are relatively common disorders and the utility of genetic testing is well established; testing has been recommended in clinical guidelines for many years [3, 4]. Initially, testing took the form of sequential Sanger sequencing of single genes, but this approach has been largely superseded in the last 5–10 years by NGS approaches that allow simultaneous testing of multiple genes in panels. More recently, WES and WGS have shown considerable promise in research and in some clinical settings, notably in unexplained infant and childhood onset disease (where de novo variants and recessive disease are common). However, consensus is yet to be reached regarding the optimal NGS approach in routine clinical care in the context of well-defined adult-onset familial disease. This is primarily due to considerable uncertainty surrounding the clinical utility of the additional data that could be provided by WES and WGS, compared to that provided by targeted panel tests.

Given this uncertainty, we designed a study that aimed to investigate which attributes of genomic testing are valued by health professionals who order genomic tests in the context of inherited cardiovascular disease. Our objective was to determine which attributes encouraged or discouraged the uptake of genomic tests in this clinical area, and whether this differed by test type.

To achieve our study aim we conducted a discrete choice experiment (DCE). A DCE is a survey-based approach that elicits the preferences of stakeholders by asking them to make trade-offs between different attributes of an intervention. DCEs usually take the form of a series of choices in which at least two alternatives are specified in terms of their attributes, which can vary across a finite number of levels. Respondents are asked to complete these choice tasks in a survey and regression analysis is then used develop a model of choice behaviour. DCEs are particularly useful in the context of genomic testing as they can provide decision-makers with insight into the relative preferences of different stakeholders for both the health and non-health benefits of testing [5–9].

In this paper, we present a DCE that was conducted amongst health professionals in the United Kingdom (UK) who order or who have ordered genetic and/or genomic tests for patients with a suspected inherited cardiovascular disease. We investigate the relative preferences of health professionals for the different attributes of genomic testing in this context, and consider how these preferences might impact on the uptake of these tests in the UK National Health Service (NHS). We also explore the factors that drive preference heterogeneity in this population.

## Materials and methods

### Sample population

The sampling population selected for this DCE was UK health professionals who order or who have ordered genetic and/or genomic tests for patients with a suspected inherited cardiovascular disease. To ensure that the sampling population reflected health professionals with a range of characteristics and preferences, respondents were recruited from two sources. First, we circulated the DCE survey amongst the members of the Genomics England Clinical Interpretation Partnership (GeCIP) in cardiovascular disease, a group of ~200 clinicians and researchers who are undertaking research on cardiovascular disease as part of the 100,000 Genomes Project in England [10]. Second, we circulated the survey amongst the members of the Association for Inherited Cardiac Conditions (AICC). At the time the survey was undertaken, the AICC had 346 members, primarily physicians, nurses, genetic counsellors or professionals allied to medicine who are actively involved in the care of patients with inherited cardiac conditions, including those with an active research interest in this area. The members of the GeCIP and the AICC are generally based in the specialist ICC centres and laboratories in the UK, and the vast majority of referrals for genetic testing in inherited cardiac conditions come from these centres.

### Defining attributes and levels for each testing alternative

Attributes and levels (the values that an attribute could take) for different genomic testing alternatives were developed using several approaches, and are described in Table 1. First, a literature review was conducted to identify studies providing information on factors related to genetic and genomic testing that are important to health professionals (see Appendix 1). From this review, 16 potential test attributes were taken forward to interviews with four health professionals (two clinicians, one laboratory scientist and one genetic counsellor) who rated the importance of each attribute when deciding whether to order genomic tests in inherited cardiovascular disease. Average importance scores were calculated and five attributes were identified that were ranked highly by all four professionals, were clinically and scientifically plausible, and could capture the key characteristics of current and future testing practice. One of these attributes was the ability of the test to identify pathogenic mutations. These changes were described to participants as disease causing variants in known genes that are clinically actionable (for example, could be used to guide treatment decisions or evaluate at risk relatives).

**Table 1:** Summary of attributes and levels used in the DCE survey

Test attribute	Possible levels for each testing alternative			
	Whole genome sequencing	Whole exome sequencing	Cardiac panel test	Genetic testing not indicated
Ability of the test to identify pathogenic mutations	Pathogenic mutation is identified in 20 out of every 100 cases			Pathogenic mutation is identified in 0 out of every 100 cases
	Pathogenic mutation is identified in 30 out of every 100 cases			
	Pathogenic mutation is identified in 40 out of every 100 cases			
	Pathogenic mutation is identified in 50 out of every 100 cases			
Ability of the test to identify variants of unknown significance	Variant of unknown significance is identified in 10 out of every 100 cases			Variant of unknown significance is identified in 0 out of every 100 cases
	Variant of unknown significance is identified in 20 out of every 100 cases			
	Variant of unknown significance is identified in 30 out of every 100 cases			
Test cost	£1000	£500	£150	£0
	£3000	£1500	£300	
	£5000	£2500	£450	
	£7000	£3500	£600	
Quantity of counselling received	40 min	40 min	10 min	0 min
	50 min	50 min	20 min	
	60 min	60 min	30 min	
Disclosure of secondary findings	No secondary findings disclosed		No secondary findings disclosed	
	Subset of well-characterised secondary findings disclosed			

Following attribute identification, the testing alternatives that would be presented to respondents in each choice task were selected. These testing alternatives were informed by the interviews with health professionals and the clinical and scientific expertise of the study team. Four testing alternatives were selected: WGS, WES, a cardiac panel test and genetic testing not indicated. This final option provided an opt-out alternative if none of the genomic testing options were viewed favourably by respondents, improving the realism of the choice tasks and allowing us to estimate the likely uptake of each alternative [11].

Levels were then identified for each attribute, informed by the interviews, literature searches and test characteristics. For some attributes, these levels were specific to each testing alternative (rather than being common to all testing alternatives). For example, the options for the cost of each testing alternative ranged from £150–£600 for the panel test, £500–£3500 for WES, and £1000–£7000 for WGS. For quantity of counselling received, we assumed that patients undergoing WES or WGS would always receive more counselling than patients undergoing panel testing (or no testing at all). We also assumed that it was not possible for a subset of well-characterised secondary findings to be disclosed if a panel test was used. Furthermore, if a subset of well-characterised secondary findings was disclosed to patients undergoing WES or WGS, we assumed that at least 50 min of counselling would be provided, based on clinical experience. The levels for the opt-out option were explicitly defined for each attribute.

### Constructing the choice tasks

In each choice task, respondents were presented with the same hypothetical situation (Box 1**)**. This situation described a patient with a phenotype suggesting inherited cardiovascular disease (but not a specific condition), which could be investigated using several testing approaches. The attribute levels for the four testing alternatives were then described (these varied in each choice question), and respondents were asked which testing option they would select for this patient. If they selected ‘Genetic testing not indicated’, they were asked which testing option they would select if this opt-out was not available. This ensured that we would generate full information on respondent preferences for genomic testing even if ‘Genetic testing not indicated’ was selected frequently. Overall, respondents were presented with 12 choice questions. This decision allowed us to achieve level balance (by attribute/testing alternative, all levels appear the same number of times across the 12 choice questions) and is in line with earlier DCEs in terms of the acceptable number of choice tasks [5, 12, 13].

### Experimental design

The experimental design of a DCE generates the combinations of attribute levels for each testing alternative that are presented in each choice task. Our experimental design was produced using Ngene (ChoiceMetrics (2018) *Ngene 1.2.0 User Manual & Reference Guide*, Australia) and is described in Appendix 2.

### Assembling the survey

The final survey was organised as follows (a copy of the survey is provided in Appendix 3). Following a welcome page, respondents were provided with information on the use of genomic testing in inherited cardiovascular disease, instructions on how to complete the survey, and information on the test attributes. Respondents were then asked to rank the attributes in terms of their relative importance when making this choice decision, and to complete a practice choice question (Box 1), designed so that WES was unequivocally the best choice that respondents could make in terms of the levels selected for each attribute/testing alternative (unless respondents had strong preferences for one of the labelled alternatives). WES was selected to be the ‘best’ choice in this practice question as we considered that respondents might have stronger preferences for or against the other labelled alternatives). Respondents then completed the 12 choice tasks and undertook a second ranking exercise to understand whether their preferences had changed during survey completion. Finally, information was collected on respondent characteristics such as experience of genomic testing, occupation and sociodemographic information.

### Piloting the survey

Two pilot surveys were conducted: one in members of the study team and local collaborators, and one in health professionals at the Oxford University Hospitals National Health Service Foundation Trust. Several presentational changes were made following these pilot surveys, and some survey text was revised for clarity. As no comments were received that necessitated revising the experimental design, the pilot survey design was retained for the main survey.

### Administering the survey

The DCE was conducted as an online survey, generated using LimeSurvey (LimeSurvey GmbH, Hamburg, Germany), between October 2017 and February 2018. Respondents in both sample populations were contacted electronically to invite them to complete the DCE. For the GeCIP, respondents were contacted by the GeCIP administrative team by email, and received two email reminders. For the AICC, respondents were recruited via an article in the AICC newsletter, and received two email reminders from the AICC administrative study team.

### Data analysis

Data analysis was undertaken using Stata (StataCorp. 2015. Stata Statistical Software: Release 14. College Station, TX: StataCorp LP). Respondent characteristics and responses to ranking questions were summarised using descriptive statistics. Observed uptake of the different testing alternatives was calculated by summarising the choices made by each respondent. These choices were also reviewed to determine whether respondents exhibited dominant preferences (i.e., frequently selected the testing alternative with the best level of a particular attribute). Further details on the dominance calculations are provided in Appendix 4.

Mixed logit regression analysis was used to construct a model of choice behaviour. This approach accommodates the existence of preference heterogeneity—which was anticipated in this sample population and clinical context—by allowing one or more model parameters to be randomly distributed across the sample population [14, 15]. In practical terms, this means that standard deviations are generated that quantify preference heterogeneity for attributes and attribute levels. Appendix 5 describes how this model was developed. In addition, because we offered respondents a choice between labelled alternatives in the choice questions, we were able to generate alternative-specific constants for WGS, WES and panel testing in our regression analysis. These constants capture the strength of respondents preferences for each choice alternative, over and above their preferences for the attributes and attribute levels that are associated with that alternative. These constants therefore capture a variety of factors, which might include respondents’ prior beliefs and perceptions of the attributes of these testing alternatives in current and future clinical practice.

In our initial analysis, we used mixed logit regression to evaluate the choices that were made by respondents in the first part of each choice question i.e., including the opt-out. We also planned a secondary analysis in which responses to the first part of each choice question were replaced by responses to the second part of each choice question, if a respondent initially selected the opt-out. This analysis was planned to be undertaken if the proportion of respondents selecting the opt-out reached 5% (see Appendix 2 for further details).

The model of choice behaviour was used to evaluate the impact of different attributes and levels on the uptake of alternative testing approaches. We also evaluated the number of correct choice predictions made by the model, calculated respondent willingness-to-pay (WTP) for different attributes of testing, estimated the marginal rates of substitution between different attributes and levels, and used the model results to predict the likely uptake of different testing alternatives. Finally, we extended this model to explore the drivers of response heterogeneity in this population by including respondent characteristics as interaction terms.

Box 1: Hypothetical choice situation presented to respondents—practice question A 44-year-old male patient presents with shortness of breath following minimal exertion. He has two children and several siblings who also have children. He has a family history of hypertrophic cardiomyopathy. An echocardiogram shows asymmetric septal hypertrophy (maximum 16 mm), confirmed by MRI scan. His coronary arteries are normal.

Three tests are available that might be able to provide genetic information that could assist with the management of this patient and his family. You can order one of these tests, or you can decide that genetic testing is not indicated for this patient.

Please review these four testing options.

**Table:** 

**Test attribute**	**Whole genome sequencing**	**Whole exome sequencing**	**Cardiac panel test**	**Genetic testing not indicated**
Ability of the test to identify pathogenic mutations	Pathogenic mutation is identified in **40** out of every **100** cases	Pathogenic mutation is identified in **50** out of every **100** cases	Pathogenic mutation is identified in **30** out of every **100** cases	Pathogenic mutation is identified in **0** out of every **100** cases
Ability of the test to identify variants of unknown significance	Variant of unknown significance is identified in **30** out of every **100** cases	Variant of unknown significance is identified in **10** out of every **100** cases	Variant of unknown significance is identified in **20** out of every **100** cases	Variant of unknown significance is identified in **0** out of every **100** cases
Test cost	£3000	£500	£600	£0
Quantity of counselling required	50 min	60 min	20 min	0 min
Disclosure of secondary findings	**No** secondary findings disclosed	**No** secondary findings disclosed	**No** secondary findings disclosed	**No** secondary findings disclosed

Which testing option would you select for this patient?

□ Whole genome sequencing

□ Whole exome sequencing

□ Cardiac panel test

□ Genetic testing not indicated

[If ‘Genetic testing not indicated’ is selected] Now suppose that ‘Genetic testing not indicated’ is not an option. Which testing option would you select for this patient now?

□ Whole genome sequencing

□ Whole exome sequencing

□ Cardiac panel test

## Results

Thirty-seven respondents completed the DCE survey. Table 2 describes their characteristics. Two-thirds of respondents were male, and the most common age group was 35–44 years. Most respondents were based in NHS Trusts in large cities, although these Trusts were located around the UK. Respondents were primarily cardiologists (40%) or clinical geneticists (31%), with the largest number qualifying between the years 1990–1999. On average, respondents saw one patient with suspected or confirmed inherited cardiovascular disease every working day, and most had previously ordered a genetic or genomic test. Most respondents (95%) thought that genomic tests for CVD patients should be made available in the NHS. Finally, respondents found the DCE survey straightforward to complete, with most completing it in less than 20 min.

**Table 2:** DCE respondent characteristics

Variable	Value	*N*
Male, *n* (%)	24 (65%)	37
Age of respondents, *n* (%)		37
25–34 years	1 (3%)	
35–44 years	15 (41%)	
45–54 years	11 (30%)	
55–64 years	9 (24%)	
65 years and over	1 (3%)	
Number of respondents working in different National Health Service Trusts, *n* (%)^a^		34
Newcastle upon Tyne Hospitals NHS Foundation Trust	5 (15%)	
Oxford University Hospital NHS Foundation Trust	4 (12%)	
Manchester University NHS Foundation Trust	3 (9%)	
Cambridge University Hospitals NHS Foundation Trust	2 (6%)	
Great Ormond Street Hospital	2 (6%)	
NHS Grampian	2 (6%)	
Others	16 (47%)	
Respondent occupation, *n* (%)^a^		35
Cardiologist	14 (40%)	
Clinical geneticist	11 (31%)	
Genetic counsellor	4 (11%)	
Academic researcher	2 (6%)	
Other	4 (11%)	
Respondent year of qualification, *n* (%)		34
1970–1979	3 (9%)	
1980–1989	9 (26%)	
1990–1999	11 (32%)	
2000–2009	9 (26%)	
2010 onwards	2 (6%)	
Respondent specialty, *n* (%)^a^		34
Cardiology related	23 (68%)	
Clinical or medical genetics	7 (21%)	
Other	4 (12%)	
Median number of patients with suspected or confirmed inherited CVD seen by respondents in a typical month^b^	20	37
Number of respondents who have ever ordered a genetic or genomic test for a patient with suspected or confirmed inherited CVD, *n* (%)^c^	35 (95%)	37
Number of respondents who have ever ordered a genetic or genomic test for a patient with another disorder, *n* (%)^c^	19 (56%)	34
Number of respondents who think genomic tests for CVD patients should be made available in the UK NHS, *n* (%)^c^	35 (95%)	37
Survey difficulty, *n* (%)		37
Very easy	6 (16%)	
Easy	9 (24%)	
Quite easy	9 (24%)	
Neither easy nor difficult	12 (32%)	
Quite difficult	1 (3%)	
Difficult	0 (0%)	
Very difficult	0 (0%)	
Median time to complete the survey, minutes^d^	15.3	37

*CVD* cardiovascular disease, *NHS* United Kingdom National Health Service, *SD* standard deviation

^a^Categories containing two or more respondents are listed. Remaining respondents are grouped under ‘Other’

^b^The mean number of patients seen in a typical month was 24. However, there was considerable variation between respondents (SD = 26.6 patients), with a small number of respondents seeing many patients in a typical month. The median value was therefore viewed as being a more appropriate measure of central tendency

^c^Further information is provided on the responses to these questions in Appendix 7

^d^The mean completion time was 23.3 min. However, there was considerable variation between respondents (SD = 28.7 min), with a small number of respondents taking a long time (likely due to pausing whilst completing the survey). The median value was therefore viewed as being a more appropriate measure of central tendency

### Ranking exercise

Appendix 6 presents the results of the ranking exercise. Both before and after completing the choice questions, the attribute that was ranked by respondents as being the most important was the ability of the test to identify pathogenic mutations.

### Model of choice behaviour

Each respondent answered 12 choice questions and there was no missing data, so 444 choices were completed in total. 21 respondents (57%) selected WES in the practice question. The remaining 16 respondents selected the panel test. Across the 444 choice questions, WGS was chosen 82 times (18% of all choices), WES was chosen 62 times (14%), the panel test was chosen 294 times (66%) and the opt-out was chosen 6 times (1%). Given this, the planned secondary analysis was not undertaken. Appendix 4 reports the results of the dominance calculations. Five respondents (14%) always selected the choice alternative that identified the most pathogenic mutations, while 4 respondents (11%) selected the panel test in every choice question (these 4 respondents also selected panel testing in the practice question).

Table 3 reports the results of the mixed logit regression analysis. The alternative-specific constants for WGS, WES and the panel test all had large positive coefficients, indicating that respondents preferred all three testing approaches compared to not testing at all (although the coefficient for WGS was not significant). The attribute coefficients indicate that uptake is more likely for tests that identify more pathogenic mutations, identify fewer variants of unknown significance, cost less, and are associated with shorter periods of counselling. Uptake might also be more likely for tests that provide information on secondary findings, but this coefficient is not significant. The standard deviations suggest that there is considerable heterogeneity around several of these parameters, including the alternative-specific constant for panel testing, the ability of the test to identify pathogenic mutations, the ability of the test to identify variants of unknown significance, and test cost. Appendix 8 presents the distributions of individual-level coefficients, illustrating the heterogeneity in these results.

**Table 3:** Mixed logit regression analysis

Attribute		Β-coefficient	SE	Lower CI	Upper CI	*P*	Willingness-to-pay	Lower CI	Upper CI
**Random parameters**									
ASC_WGS	Mean	3.194	2.700	−2.097	8.486	0.237	£486.27	−£198.45	£1170.98
	SD	0.386	0.758	−1.099	1.871	0.610	–	–	–
ASC_PANEL	Mean	4.851	2.071	0.791	8.911	0.019	£738.52	£300.39	£1176.65
	SD	4.254	1.196	1.910	6.599	0.000	–	–	–
PATHOGENIC	Mean	0.768	0.238	0.301	1.235	0.001	£116.90	£99.14	£134.65
	SD	0.797	0.290	0.229	1.365	0.006	–	–	–
UNKNOWN	Mean	−0.213	0.083	−0.375	−0.050	0.010	−£32.38	−£48.22	−£16.53
	SD	0.304	0.110	0.087	0.520	0.006	–	–	–
COST	Mean	−0.007	0.002	−0.011	−0.002	0.003	–	–	–
	SD	0.015	0.005	0.005	0.025	0.003	–	–	–
Fixed parameters									
ASC_WES	Mean	5.879	2.944	0.109	11.649	0.046	£894.94	£280.22	£1509.66
COUNSEL	Mean	−0.097	0.043	−0.181	−0.012	0.025	−£14.72	−£24.30	−£5.15
SECONDARY	Mean	−0.052	0.474	−0.981	0.876	0.912	−£7.95	−£148.17	£132.26
McFadden’s *R*^2^ (pseudo *R*^2^)	0.24								
AIC	366.76								
Log-likelihood	−160.38								

Attribute descriptions: ASC = alternative-specific constant for whole genome sequencing, whole exome sequencing or the cardiac panel test; PATHOGENIC = ability of the test to identify pathogenic mutations; UNKNOWN = ability of the test to identify variants of unknown significance; COST = test cost; COUNSEL = quantity of counselling received; SECONDARY = disclosure of secondary findings.

*AIC* akaike information criterion*, CI*  95% confidence interval,  *P* *p* value, *SD* standard deviation, *SE* standard error

The ratios between the different attribute coefficients provide information on the trade-offs that respondents were willing to make. For a given test, respondents would be willing to tolerate a 36% increase in VUS if the proportion of pathogenic mutations detected increases by 10%. In terms of test uptake, respondents would require a pathogenic mutation detection rate of at least 6.3% before adopting panel testing instead of no testing, and would require a mutation detection rate of at least 7.7% before adopting WES instead of no testing.

Table 3 also reports WTP estimates. Respondents were willing to pay £739 for panel testing or £895 for WES if the alternative in both cases was no testing at all. Respondents were also willing to pay £117 for every 1% increase in the pathogenic mutation detection rate, and £32 for every 1% decrease in the detection rate for variants of unknown significance. Finally, respondents were willing to pay £15 for every 1 min reduction in counselling provided.

Overall, this model of choice behaviour correctly predicts 433 of the 444 (98.8%) hypothetical choices made by respondents.

### Test uptake

To understand the likely uptake of these testing alternatives in current practice, levels were set for each test that reflected current knowledge of test attributes. These levels are defined in Table 4, along with estimated utility scores for each testing alternative and estimates of the probability of uptake. Appendix 9 provides the reasoning for these assumptions and reports the calculations that underlie these estimates. Panel testing had the highest predicted uptake rate at 98% i.e., almost universal uptake.

**Table 4:** Predicted uptake of the four testing alternatives in current practice

Test	Test attributes and levels					Utility score		Probability of uptake	
	Pathogenic mutation identified in X out of every 100 cases	VUS identified in X out of every 100 cases	Cost	Counselling	Secondary findings disclosed	Mean	SD	Mean	SD
WGS	50	55	£5000	60 min	Subset	−18.5	30.8	0.10%	0.60%
WES	45	35	£2500	60 min	Subset	9.0	15.2	1.40%	4.10%
Panel	40	15	£300	30 min	None	33.9	15.9	98.10%	6.20%
None	0	0	£0	0 min	None	−0.1	0.0	0.40%	2.30%

*Panel* panel test, *SD* standard deviation, *VUS* variants of unknown significance, *WES* whole exome sequencing, *WGS* whole genome sequencing

### Extending the model to explore the drivers of response heterogeneity

Appendix 10 reports the methods and results of this analytical extension. Three respondent characteristics showed some potential in terms of driving the heterogeneity that was observed in respondent preferences: the number of patients with suspected or confirmed inherited cardiovascular disease that respondents saw in a typical month, respondent gender and respondent year of qualification. However, we were unable to develop a model of choice behaviour that included these characteristics.

## Discussion

We have presented the results of a DCE that was conducted in health professionals in the UK who order or who have ordered genetic and/or genomic tests for patients with a suspected inherited cardiovascular disease. Respondents had strong preferences for either WGS, WES or panel testing in this population, if the alternative was no testing at all. Irrespective of the type of test, uptake of genomic testing was positively affected by an improvement in the pathogenic mutation detection rate, a fall in the proportion of cases in which variants of unknown significance are identified, a reduction in test cost and a reduction in the amount of counselling that patients receive. Considerable heterogeneity was observed around preferences for several of these test attributes. Overall, when the current specifications of different types of genomic test were used as inputs into our model of choice behaviour, panel testing had the highest predicted uptake rate. This suggests either scepticism or lack of awareness of the benefits of WES and WGS, or an awareness of the limitations of WES and WGS.

One notable finding was that the monetary value attached to the change in the pathogenic mutation detection rate when moving from the current specification of panel testing to WES (£585) was far lower than the likely increase in cost when moving between these two tests (£2200—see Table 4). This was also true when moving from panel testing to WGS (or WES to WGS), which suggests that WES and WGS may not offer good value for money at present in this clinical context, compared to panel testing. A second notable finding was that respondents preferred tests that identify fewer variants of unknown significance. This likely reflects the fact that when WES or WGS are undertaken in the context of an adult-onset autosomal dominant disorder, VUSs are both common and also unlikely to convert to variants that affect function over time. A further finding was that a reduction in the amount of counselling that patients receive had a positive effect on the uptake of genomic testing. This may be an artefact of the design of the DCE. Specifically, panel testing was strongly favoured by respondents, and the quantity of counselling received by patients undergoing panel testing was constrained by the experimental design to always be less than the quantity of counselling received by patients undergoing WES or WGS. That said, the relative size of the coefficient for the counselling attribute in our regression suggests that this attribute likely only had a small impact on respondent choices.

These findings match those of other DCEs that have evaluated the preferences of health professionals, albeit in the context of treatment rather than genetic testing. A common finding is that health professionals place greater value on structure and outcome attributes (such as the mutation detection rate) than on process outcomes (such as the amount of counselling that patients receive). The preferences indicated by respondents in this DCE support this conclusion [16].

This is one of the first studies to evaluate the preferences of those stakeholders who could be instrumental in the translation of genomic tests into clinical practice in a number of clinical disciplines. Given this, our results may be generalisable to other contexts in which there is clinical equipoise regarding the health and non-health benefits of genomic testing, in particular in clinical settings where well-defined, autosomal dominant disorders predominate. However, a number of caveats should be noted. First, in our DCE, respondents completed choice questions whilst considering a single hypothetical scenario, which was designed to be deliberately borderline. Alternative scenarios might generate a different pattern of results regarding test uptake. Second, we observed considerable preference heterogeneity but were unable to explore this in depth due to the sample size of our study. The drivers of preference heterogeneity in health professionals regarding genomic testing in this context should be explored further in qualitative studies before extrapolating these results to other clinical contexts. Such studies could also help to explain why respondents selected panel testing in the practice question instead of WES. Revealed preference studies, which generate information on how respondents actually behave, rather than how they say they will behave, would also help to explain this finding. The imminent rollout of the UK NHS Genomic Medicine Service may present an opportunity to collect such revealed preference data [17].

A number of additional limitations should be noted. First, we were not able to calculate a response rate for this DCE survey because it was not possible to precisely determine either the number of members of the GeCIP, or the number of members of either sampling population that were responsible for ordering genetic and/or genomic tests for patients with a suspected inherited cardiovascular disease. Second, our experimental design used alternative-specific attribute levels. This meant that we could not achieve level balance for all attributes, which had a negative impact on the statistical efficiency of our study design. The use of a labelled design had the same impact. However, in both cases these design decisions reduced hypothetical bias, which meant that the choice scenarios were likely more realistic for respondents, improving response efficiency [18]. The overall impact of these design decisions is therefore unclear. Third, we asked respondents to complete an attribute ranking exercise before and after completing the choice questions. As respondents were not asked to consider the type of genomic test in this exercise, it is not possible to fully corroborate the DCE results by comparing them with those of the ranking exercise. Fourth, a larger sample size would have allowed us to explore the drivers of respondent heterogeneity in more depth, and might have yielded more reliable estimates of attribute coefficients. However, it has been previously noted that a sample size of twenty is sufficient to reliably estimate a DCE model, given that each respondent provides multiple observations [19]. Furthermore, we believe that our sample includes many of the health professionals who order genetic and genomic tests in this clinical specialty in the UK. Fifth, one of the attributes in our DCE was the ability of the test to identify pathogenic mutations. It is now recommended that the term “variant” is used instead of “mutation” to avoid confusion [20]. No respondents raised concerns regarding the use of this term in the free text comments. Finally, our estimates of willingness-to-pay were generated by evaluating the marginal rate of substitution between cost and the other test attributes. However, an alternative approach—estimating willingness-to-pay in willingness-to-pay space rather than preference space—might be more valid when a cost attribute is included in a mixed logit regression as a random parameter [15].

## Conclusion

The use of genomic tests in patients selected for variant analysis in the context of inherited cardiovascular disease is valued by health professionals. Our study found that uptake of these tests is more likely if the pathogenic mutation rate increases, fewer variants of unknown significance are identified, or if they decrease in cost. However, the health professionals who responded to this survey have strong preferences for a specific type of genomic test—panel testing—rather than WES and WGS. Such preferences will be influenced by the genetic architecture of the diseases that predominate in each medical specialty; the yield of pathogenic mutations is likely to always be a principal driver of test uptake, but where disease is caused by single heterozygous variants, high VUS yield may limit uptake of WES and WGS. These preferences may impact on the translation of WES and WGS into clinical practice for heterozygous adult diseases, both in the UK and internationally, in the short-to-medium term. This finding has implications for the design of future genomic testing services, and suggests that different uptake rates should be explicitly modelled when estimating the cost-effectiveness of alternative testing strategies. Given the preference heterogeneity observed in the context of inherited cardiovascular disease, similar studies specifically relating to genetic testing in other medical specialties would contribute significantly to the debate surrounding the translation of WES and WGS into routine clinical practice.

## Supplementary information

Supplementary materials
