# Supplementary material for: Do health professionals value genomic testing? A discrete choice experiment in inherited cardiovascular disease
Source: Eur J Hum Genet. 2019 Jun 11;27(11):1639–48. doi: 10.1038/s41431-019-0452-z (PMC6870981; doi:10.1038/s41431-019-0452-z)
Supplement: Supplementary file 1 — Supplementary materials [file 41431_2019_452_MOESM1_ESM.docx]

**Do health professionals value genomic testing? A discrete choice experiment in inherited cardiovascular disease**

**Supplementary materials**

James Buchanan MA DPhil ^1,2*^, Edward Blair BMSc MBchB ^3,4^, Kate L Thomson BSc FRCPath ^5,6^, Elizabeth Ormondroyd MSc PhD ^2,7^, Hugh Watkins MD PhD ^2,7,8^, Jenny C Taylor MA DPhil ^2,7^, Sarah Wordsworth MSc PhD ^1,2^

* Corresponding author: [james.buchanan@dph.ox.ac.uk](mailto:james.buchanan@dph.ox.ac.uk), +44 1865 289262

1. Health Economics Research Centre, Nuffield Department of Population Health, University of Oxford, Old Road Campus, Oxford, OX3 7LF, UK
2. National Institute for Health Research Biomedical Research Centre, Oxford, UK
3. Oxford Centre for Genomic Medicine, Oxford University Hospitals NHS Foundation Trust, Oxford, UK
4. Department of Clinical Genetics, Churchill Hospital, Headington, Oxford, UK
5. Oxford Medical Genetics Laboratory, Oxford University Hospitals NHS Foundation Trust, The Churchill Hospital, Oxford, UK
6. Radcliffe Department of Medicine, University of Oxford, Oxford, UK
7. Division of Cardiovascular Medicine, Radcliffe Department of Medicine, University of Oxford, Oxford, UK
8. Wellcome Trust Centre for Human Genetics, University of Oxford, UK

**Contents**

[Appendix 1: Literature review 2](#_Toc531124076)

[Appendix 2: Experimental design 3](#_Toc531124077)

[Appendix 3: Final DCE survey 5](#_Toc531124078)

[Appendix 4: Dominance calculations and results 34](#_Toc531124079)

[Appendix 5: Development of the mixed logit model of choice behaviour 36](#_Toc531124080)

[Appendix 6: Attribute rankings, before and after completing the choice questions 37](#_Toc531124081)

[Appendix 7: Further information on respondent use of genetic and genomic tests, and on their preferences regarding the future use of genomic testing 38](#_Toc531124082)

[Appendix 8: Distributions of individual-level coefficients 42](#_Toc531124083)

[Appendix 9: Observed and predicted uptake probabilities 45](#_Toc531124084)

[Appendix 10: Extending the model to explore the drivers of response heterogeneity 48](#_Toc531124085)

[References 49](#_Toc531124086)

# Appendix 1: Literature review

Methods

A literature review was conducted to identify papers that provide information on those factors related to WGS that are important to clinicians. A search strategy was designed for Scopus, with the following syntax:

TITLE-ABS-KEY("discrete choice *" OR DCE OR "conjoint analysis" OR "best-worst *" OR BWS OR attributes OR "* preferences") AND TITLE-ABS-KEY("cancer screening" OR "screening test" OR "treatment focused" OR "next-generation sequencing" OR "third-generation sequencing" OR "whole * sequencing" OR "targeted treatment" OR "gen* testing" OR "gen* sequencing" OR "pharmacogen* testing" OR "personali* medicine" OR "tumour profiling" OR "DNA sequencing" OR "molecular diagnos*" OR "cancer panel")

Papers were included in the review if they were:

- Published from 2004 onwards;
- Written in English;
- Publications in the areas of medicine, pharmaceuticals, nursing, social sciences, health professions, psychology, neuroscience, economics, or decision sciences;
- Journal articles, reviews or articles in press.

The literature search was undertaken in November 2015. Following deduplication, publications were scanned for relevance by title and abstract, and clearly irrelevant publications were excluded. The full text of the remaining publications was then evaluated for relevance. Information was extracted from included publications on factors related to WGS that are important to clinicians.

Results

The database search identified 1,861 papers. After removing duplicates, screening titles and abstracts, and then assessing full-text papers for eligibility, 153 papers were identified that met the inclusion criteria. A review of the reference lists of retrieved publications identified 8 additional relevant publications. Overall, 161 papers were included in the review.

107 unique factors related to WGS were identified in these papers. Of these factors, 31 appeared in only 1 paper, whereas 28 appeared in 10 or more papers. 16 factors were identified that might be potentially important to clinicians. These were taken forward to the interviews with health professionals.

# Appendix 2: Experimental design

The aim of the DCE was to model the probability that a health professional would select a specific genomic test. The probability of choosing a given alternative is determined by the indirect utility (*V*) associated with that alternative. The indirect utility function differed for the two parts of each choice task that we presented to respondents. In the first part of each choice task, we presented all three test options and the opt-out alternative, so the indirect utility function was:

*V* = α_1_.ASC_WGS_ + α_2_.ASC_WES_ + α_3_.ASC_PANEL_ + β_1_.PATHOGENIC + β_2_.UNKNOWN + β_3_.COST + β_4_.COUNSEL + β_5_.SECONDARY + γ.Z + ε

Where: α_1_ to α_3_ = estimates for the alternative-specific constants (ASCs) for the three test options

β _1_ to β_5_ = parameters

Z = set of covariate terms of interest

γ = set of estimates for these covariate terms

ε = error term (unobservable random component)

PATHOGENIC = the ability of a test to identify pathogenic mutations

UNKNOWN = the ability of a test to identify variants of unknown significance

COST = the cost of a test

COUNSEL = the quantity of counselling received by the patient

SECONDARY = the level of disclosure of secondary findings

For the second part of each choice task, only the three non opt-out choice alternatives were presented to respondents. The indirect utility function was therefore:

*V* = α_1_.ASC_WGS_ + α_2_.ASC_WES_ + β_1_.PATHOGENIC + β_2_.UNKNOWN + β_3_.COST + β_4_.COUNSEL + β_5_.SECONDARY + γ.Z + ε

The expected impact of each of the attributes on utility was:

| **Attribute** | **Expected impact on utility of an increase in the attribute** |
| --- | --- |
| PATHOGENIC | Positive |
| UNKNOWN | Negative |
| COST | Negative |
| COUNSEL | Positive |
| SECONDARY | Unclear ^a^ |

^a^ It was unclear whether respondents would want to receive information on secondary findings, hence the impact of receiving information on a subset of findings is not necessarily positive

Four of the five attributes were assumed to be linear and thus coded as continuous variables. The exception was SECONDARY, for which effects-coding was used.

The two-part nature of the choice tasks was accommodated in the experimental design by using a model averaging approach. Ngene (ChoiceMetrics (2018) *Ngene 1.2.0 User Manual & Reference Guide*, Australia) was used to generate designs for both parts of the choice tasks that were then combined to generate an overall d-efficient design. In this combined design, the design for the second part of the choice task (excluding the opt-out) was given a weighting of 0.05, and the design for the first part of the choice task was given a weighting of 0.95. This reflected the belief of the study team that only 5% of respondents would select the opt-out across all choice tasks, given the hypothetical alternative that was presented to respondents.

The final combined design minimised the d-error for a multinomial logit (MNL) model with no dominated alternatives and minimal overlap. This design also exhibited level balance for most attributes. The exception was the levels for the COUNSEL and SECONDARY attributes. The imbalance on these attributes for the WGS and WES alternatives arose because of the constraint that was applied on counselling time when a subset of well-characterised secondary findings was disclosed to patients.

Given that the pilot survey did not yield information that could be used to inform the selection of priors for this experimental design, the same design was used in both the pilot survey and the main survey. The choices that were generated by this experimental design are described in **Appendix 3**.

# Appendix 3: Final DCE survey

**WELCOME PAGE**

Welcome to this survey about the use of genomic testing in inherited cardiovascular disease in the UK NHS. You have been invited to participate as you are a healthcare professional who orders or has ordered genetic and/or genomic tests for patients selected for mutation analysis in the context of inherited cardiovascular disease.

This survey is being run by the [Health Economics Research Centre](https://www.herc.ox.ac.uk) at the University of Oxford. We are interested in your views about how useful genomic testing is in this clinical context. Your participation in this survey is entirely voluntary, but your views will help to develop this testing service in the future.

Please try to answer all of the questions in this survey. There are no ‘correct’ answers – we are just interested in your views. To help you to answer the questions we have provided some background information at the start of the survey. Please read this carefully before you complete the survey.

This survey should take around 20 minutes to complete. Please click on the ‘Submit’ button at the end of the survey to ensure that your responses are included in our study. You may withdraw at any point during the survey for any reason, before submitting your answers, by closing your browser window.

All of the information that you provide will remain strictly confidential, will be anonymised and will only be used for research purposes. Your responses will be stored in a password-protected file and may be used in academic publications. The University of Oxford is the data controller for the purposes of the Data Protection Act 1998. Your information will not be shared with any other institutions.

This project has been reviewed by, and received ethics clearance through, the University of Oxford Central University Research Ethics Committee [R51851/RE001].

**What if there is a problem?**

If you have a concern about any aspect of this project, please contact Dr James Buchanan using the contact details below. Dr Buchanan will acknowledge your concern within 10 working days and give you an indication of how he intends to deal with it.

**Dr James Buchanan**

Senior Researcher

Health Economics Research Centre

Nuffield Department of Population Health

University of Oxford

Old Road Campus

Headington

Oxford

OX3 7LF

Telephone: 01865 289262

Email: [james.buchanan@dph.ox.ac.uk](mailto:james.buchanan@dph.ox.ac.uk)

If you remain unhappy or wish to make a formal complaint, please contact the Chair of the Medical Sciences Inter-Divisional Research Ethics Committee at the University of Oxford (Email: ethics@medsci.ox.ac.uk; Address: Research Services, University of Oxford, Wellington Square, Oxford OX1 2JD). The Chair will seek to resolve the matter in a reasonably expeditious manner.

**THANK YOU FOR YOUR HELP**

**CONSENT**

Having read the information on the previous page, do you agree to participate in this study?

[Tickbox] Yes, I agree to take part

**BACKGROUND**

There are a wide range of cardiovascular conditions with an inherited component.

Some of these are **multifactorial** (involving interactions between multiple gene products and environmental factors) e.g. coronary artery disease, whereas others have a primarily **monogenic** cause (involving a single gene) e.g. hypertrophic cardiomyopathy.

Many genes are known to be associated with monogenic inherited cardiac conditions. Genetic tests are increasingly available for the common monogenic cardiac disorders, providing information that can be used to guide evaluation of relatives and, rarely, influence the clinical management of patients. These tests are now recommended by both national and international clinical guidelines, and have become an integral part of clinical and family management in many centres.

**Genomic tests**

Attention is now turning towards the use of **genomic tests** that evaluate multiple genes simultaneously.

These tests can provide information that informs the management of patients and families with either monogenic or multifactorial cardiac disorders.

Several types of genomic test could be useful in this clinical context, including:

- **Cardiac panel tests** (which test a subset of key well established genes known to cause inherited cardiac conditions);
- **Whole exome sequencing** (which permits the analysis of genes translated into proteins; the ‘exome’), and
- **Whole genome sequencing** (which permits the analysis of the entire DNA sequence; the ‘genome’ of an individual).

Cardiac panel tests are now in routine use in the UK NHS, and whole exome and whole genome sequencing are also becoming available.

The aim of this survey is to learn more about the preferences of healthcare professionals for these tests. This will help us to understand which test attributes are most important, what the potential barriers are to their use in the NHS, and what the likely uptake is.

**HOW TO COMPLETE THIS SURVEY**

Please consider the following hypothetical situation:

*A 44 year old male patient presents with shortness of breath following minimal exertion. He has two children and several siblings who also have children. He has a family history of hypertrophic cardiomyopathy. An echocardiogram shows asymmetric septal hypertrophy (maximum 16mm), confirmed by MRI scan. His coronary arteries are normal.*

Three tests are available that might be able to provide genetic information that could assist with the management of this patient and his family: **whole genome sequencing**, **whole exome sequencing** and a **cardiac panel test**. You can order one of these tests, or you can decide that **genetic testing is not indicated** for this patient.

In this survey you will be presented with several scenarios in which we will ask you to choose one of these three testing options (or no testing) for the patient described above. **The description of the patient is the same in each of these scenarios**. Even if you do not like any of these testing options, please indicate the option you would prefer if you had to choose one.

Each test will be described to you in terms of five test attributes:

- Ability of the test to identify pathogenic mutations
- Ability of the test to identify variants of unknown significance
- Test cost
- Quantity of counselling required
- Disclosure of secondary findings

Each attribute can take one of several levels, and these levels will vary in each scenario. The levels presented for each test in the different scenarios do not necessarily reflect current test characteristics, but instead reflect hypothetical future specifications of this test. In some cases, these levels are specific to a certain type of test (e.g. whole genome sequencing). In other cases, these levels are possible for more than one of the testing options.

Detailed descriptions of each test attribute and the possible levels are provided on the following pages. Please read these descriptions carefully, then try to answer all of the questions that follow.

**THANK YOU FOR YOUR HELP**

**TEST ATTRIBUTES – ATTRIBUTE 1**

**Ability of the test to identify pathogenic mutations**

The first attribute describes the performance of the test in terms of the proportion of cases in which a pathogenic mutation is identified.

These are variants in known genes that are clinically actionable, i.e. the test result can be used to modify patient and/or family management.

For example, a test result could be deemed clinically actionable if it can be used to guide treatment decisions or to evaluate at risk relatives. A test result that has diagnostic implications could also be considered to be clinically actionable.

For whole genome sequencing, whole exome sequencing and the cardiac panel test, the levels for this attribute range from **20** out of every **100** cases to **50** out of every **100** cases.

If genetic testing is not indicated, a pathogenic mutation is never identified (**0** out of every **100** cases).

**TEST ATTRIBUTES – ATTRIBUTE 2**

**Ability of the test to identify variants of unknown significance**

The second attribute describes the performance of the test in terms of the proportion of cases in which a variant of unknown significance is identified.

These are variants in genes that are not clinically actionable due to a lack of evidence, i.e. the pathogenicity of the variant can neither be confirmed nor ruled out.

For whole genome sequencing, whole exome sequencing and the cardiac panel test, the levels for this attribute range from **10** out of every **100** cases to **30** out of every **100** cases.

If genetic testing is not indicated, variants of unknown significance are never identified (**0** out of every **100** cases).

**TEST ATTRIBUTES – ATTRIBUTE 3**

**Test cost**

The third attribute describes the cost of testing a singleton (the proband: the patient or member of the family that brings a family under study).

This cost is incurred by the UK National Health Service (NHS) and varies between **£1,000-£7,000** for whole genome sequencing, **£500-£3,500** for whole exome sequencing and **£150-£600** for the cardiac panel test. There is no cost (**£0**) if genetic testing is not indicated.

Further costs may be incurred by the NHS following proband testing, depending on the actions taken based on the test results. For example, cascade testing may be undertaken. These costs are not quantified within the different choice scenarios.

**TEST ATTRIBUTES – ATTRIBUTE 4**

**Quantity of counselling required**

The fourth attribute describes how much counselling (in minutes) is required by the patient undergoing testing, during the whole testing process.

This includes the period before testing and the period after testing, when results are returned to the patient.

For whole genome sequencing and whole exome sequencing, the levels for this attribute range from **40** minutes to **60** minutes.

For the cardiac panel test, the levels range from **10** minutes to **30** minutes.

If genetic testing is not indicated, no counselling (**0** minutes) is provided.

**TEST ATTRIBUTES – ATTRIBUTE 5**

**Disclosure of secondary findings**

The fifth attribute describes whether any secondary findings are disclosed to the patient alongside the main test result.

Secondary findings are genomic changes that are not related to the condition that led to the patient initially undergoing testing, but which are known to cause serious, life threatening conditions that can sometimes be prevented or reduced by NHS treatment.

There are two levels for this attribute for whole genome sequencing and whole exome sequencing: **no** secondary findings are disclosed, or a **subset** of well-characterised secondary findings are disclosed (i.e. findings that are most likely to be clinically actionable).

For the cardiac panel test, and when genetic testing is not indicated, the only option is that **no** secondary findings are disclosed.

**TEST ATTRIBUTES - SUMMARY**

**Summary of test attributes and levels**

The following table summarises the five test attributes, and the different levels that these attributes can take for each of the four testing options.

Please carefully review the information in this table before proceeding.

| **Test attribute** | **Possible levels** | | | |
| --- | --- | --- | --- | --- |
|  | **Whole genome sequencing** | **Whole exome sequencing** | **Cardiac panel test** | **Genetic testing not indicated** |
| **Ability of the test to identify pathogenic mutations** | Pathogenic mutation is identified in **20** out of every **100** cases | | | Pathogenic mutation is identified in **0** out of every **100** cases |
|  | Pathogenic mutation is identified in **30** out of every **100** cases | | |  |
|  | Pathogenic mutation is identified in **40** out of every **100** cases | | |  |
|  | Pathogenic mutation is identified in **50** out of every **100** cases | | |  |
| **Ability of the test to identify variants of unknown significance** | Variant of unknown significance is identified in **10** out of every **100** cases | | | Variant of unknown significance is identified in **0** out of every **100** cases |
|  | Variant of unknown significance is identified in **20** out of every **100** cases | | |  |
|  | Variant of unknown significance is identified in **30** out of every **100** cases | | |  |
| **Test cost** | £1,000 | £500 | £150 | £0 |
|  | £3,000 | £1,500 | £300 |  |
|  | £5,000 | £2,500 | £450 |  |
|  | £7,000 | £3,500 | £600 |  |
| **Quantity of counselling required** | 40 minutes | | 10 minutes | 0 minutes |
|  | 50 minutes | | 20 minutes |  |
|  | 60 minutes | | 30 minutes |  |
| **Disclosure of secondary findings** | **No** secondary findings disclosed | | **No** secondary findings disclosed | |
|  | **Subset** of well-characterised secondary findings disclosed | |  |  |

**ATTRIBUTE RANKING EXERCISE**

Before you answer the main survey questions we would like to know which of these attributes is most/least important to you.

Please consider all five test attributes and then rank them below.

Double-click or drag-and-drop items in the "Your choices" list to move them into the "Your ranking" list - your highest ranking (most important) item should be at the top of the list, moving through to your lowest ranking (least important) item at the bottom of the list.

If you would like to read the attribute descriptions again, please go back by clicking on the 'Previous' button.

[RANKING TABLE]

**PRACTICE QUESTION**

Now we would like you to complete a practice choice question.

Consider the following hypothetical situation. This is the same patient description that we previously presented to you.

*A 44 year old male patient presents with shortness of breath following minimal exertion. He has two children and several siblings who also have children. He has a family history of hypertrophic cardiomyopathy. An echocardiogram shows asymmetric septal hypertrophy (maximum 16mm), confirmed by MRI scan. His coronary arteries are normal.*

Three tests are available that might be able to provide genetic information that could assist with the management of this patient and his family. You can order one of these tests, or you can decide that genetic testing is not indicated for this patient.

Please review these four testing options.

| **Test attribute** | **Whole genome sequencing** | **Whole exome sequencing** | **Cardiac panel test** | **Genetic testing not indicated** |
| --- | --- | --- | --- | --- |
| **Ability of the test to identify pathogenic mutations** | Pathogenic mutation is identified in **40** out of every **100** cases | Pathogenic mutation is identified in **50** out of every **100** cases | Pathogenic mutation is identified in **30** out of every **100** cases | Pathogenic mutation is identified in **0** out of every **100** cases |
| **Ability of the test to identify variants of unknown significance** | Variant of unknown significance is identified in **30** out of every **100** cases | Variant of unknown significance is identified in **10** out of every **100** cases | Variant of unknown significance is identified in **20** out of every **100** cases | Variant of unknown significance is identified in **0** out of every **100** cases |
| **Test cost** | £3,000 | £500 | £600 | £0 |
| **Quantity of counselling required** | 50 minutes | 60 minutes | 20 minutes | 0 minutes |
| **Disclosure of secondary findings** | **No** secondary findings disclosed | **No** secondary findings disclosed | **No** secondary findings disclosed | **No** secondary findings disclosed |

Which testing option would you select for this patient?

| Whole genome sequencing |  |
| --- | --- |
| Whole exome sequencing |  |
| Cardiac panel test |  |
| Genetic testing not indicated |  |

[If ‘Genetic testing not indicated’ is selected]

Now suppose that ‘Genetic testing not indicated’ is not an option. Which testing option would you select for this patient now?

| Whole genome sequencing |  |
| --- | --- |
| Whole exome sequencing |  |
| Cardiac panel test |  |

**MAIN QUESTIONS**

Thank you for completing the ranking exercise and practice question.

Now we would like you to complete the main part of the survey.

We are going to present you with 12 scenarios that are similar to the practice question.

**Remember, the description of the patient is the same as the description that we presented to you in the practice question, and is the same in each of the scenarios**. However, the levels for each of the attributes will vary in each scenario.

Even if you do not like any of the testing options, please indicate the option you would select if you had to choose one.

**QUESTION 1**

Consider the following hypothetical situation.

*A 44 year old male patient presents with shortness of breath following minimal exertion. He has two children and several siblings who also have children. He has a family history of hypertrophic cardiomyopathy. An echocardiogram shows asymmetric septal hypertrophy (maximum 16mm), confirmed by MRI scan. His coronary arteries are normal.*

Three tests are available that might be able to provide genetic information that could assist with the management of this patient and his family. You can order one of these tests, or you can decide that genetic testing is not indicated for this patient.

Please review these four testing options.


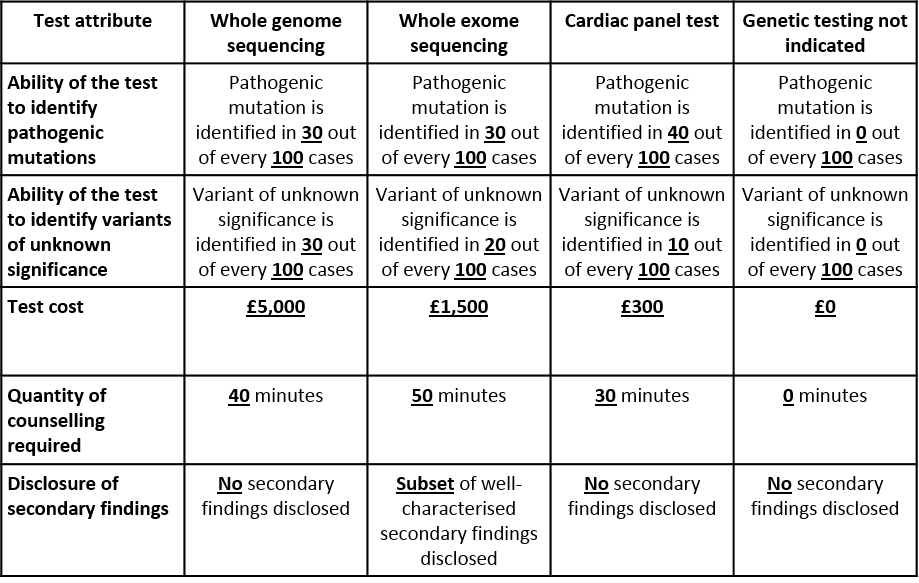


Which testing option would you select for this patient?

| Whole genome sequencing |  |
| --- | --- |
| Whole exome sequencing |  |
| Cardiac panel test |  |
| Genetic testing not indicated |  |

[If ‘Genetic testing not indicated’ is selected]

Now suppose that ‘Genetic testing not indicated’ is not an option. Which testing option would you select for this patient now?

| Whole genome sequencing |  |
| --- | --- |
| Whole exome sequencing |  |
| Cardiac panel test |  |

**QUESTION 2**

Consider the following hypothetical situation.

*A 44 year old male patient presents with shortness of breath following minimal exertion. He has two children and several siblings who also have children. He has a family history of hypertrophic cardiomyopathy. An echocardiogram shows asymmetric septal hypertrophy (maximum 16mm), confirmed by MRI scan. His coronary arteries are normal.*

Three tests are available that might be able to provide genetic information that could assist with the management of this patient and his family. You can order one of these tests, or you can decide that genetic testing is not indicated for this patient.

Please review these four testing options.


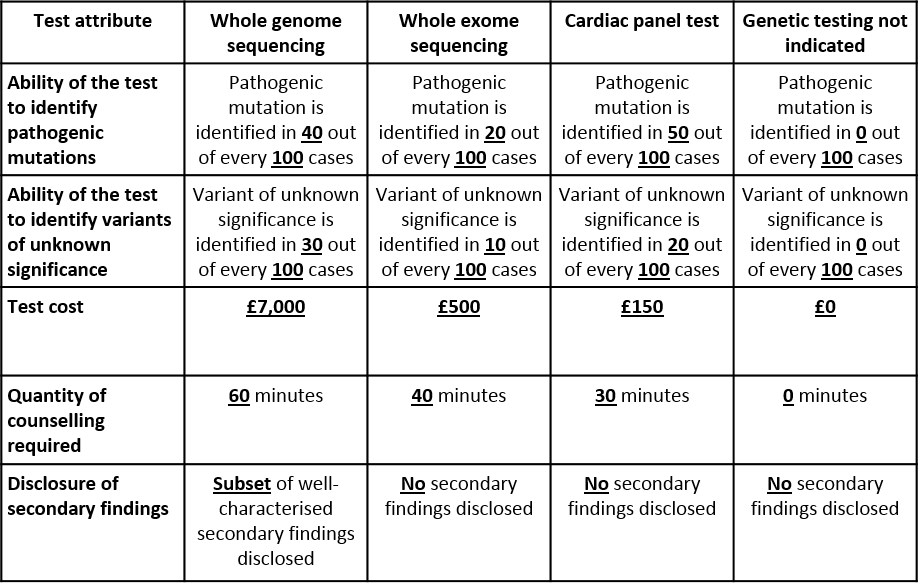


Which testing option would you select for this patient?

| Whole genome sequencing |  |
| --- | --- |
| Whole exome sequencing |  |
| Cardiac panel test |  |
| Genetic testing not indicated |  |

[If ‘Genetic testing not indicated’ is selected]

Now suppose that ‘Genetic testing not indicated’ is not an option. Which testing option would you select for this patient now?

| Whole genome sequencing |  |
| --- | --- |
| Whole exome sequencing |  |
| Cardiac panel test |  |

**QUESTION 3**

Consider the following hypothetical situation.

*A 44 year old male patient presents with shortness of breath following minimal exertion. He has two children and several siblings who also have children. He has a family history of hypertrophic cardiomyopathy. An echocardiogram shows asymmetric septal hypertrophy (maximum 16mm), confirmed by MRI scan. His coronary arteries are normal.*

Three tests are available that might be able to provide genetic information that could assist with the management of this patient and his family. You can order one of these tests, or you can decide that genetic testing is not indicated for this patient.

Please review these four testing options.


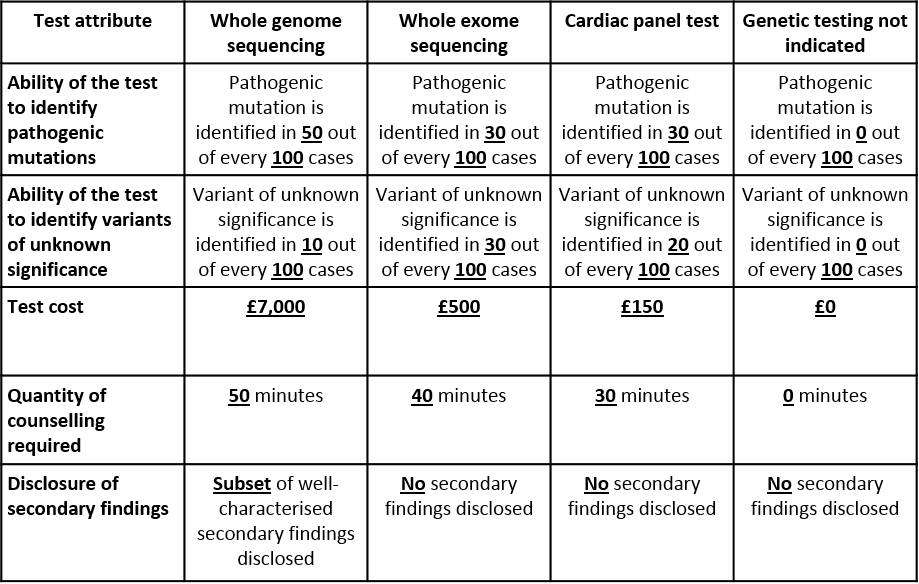


Which testing option would you select for this patient?

| Whole genome sequencing |  |
| --- | --- |
| Whole exome sequencing |  |
| Cardiac panel test |  |
| Genetic testing not indicated |  |

[If ‘Genetic testing not indicated’ is selected]

Now suppose that ‘Genetic testing not indicated’ is not an option. Which testing option would you select for this patient now?

| Whole genome sequencing |  |
| --- | --- |
| Whole exome sequencing |  |
| Cardiac panel test |  |

**QUESTION 4**

Consider the following hypothetical situation.

*A 44 year old male patient presents with shortness of breath following minimal exertion. He has two children and several siblings who also have children. He has a family history of hypertrophic cardiomyopathy. An echocardiogram shows asymmetric septal hypertrophy (maximum 16mm), confirmed by MRI scan. His coronary arteries are normal.*

Three tests are available that might be able to provide genetic information that could assist with the management of this patient and his family. You can order one of these tests, or you can decide that genetic testing is not indicated for this patient.

Please review these four testing options.


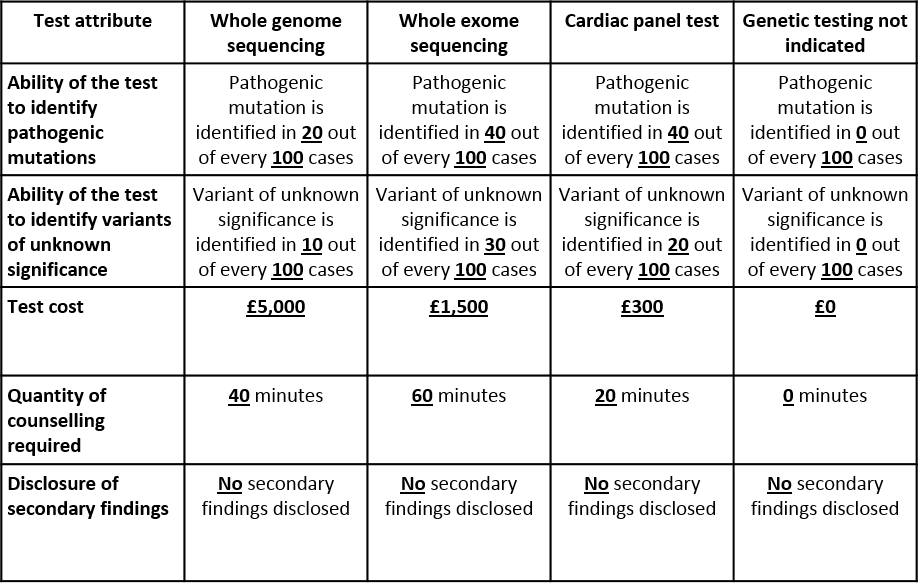


Which testing option would you select for this patient?

| Whole genome sequencing |  |
| --- | --- |
| Whole exome sequencing |  |
| Cardiac panel test |  |
| Genetic testing not indicated |  |

[If ‘Genetic testing not indicated’ is selected]

Now suppose that ‘Genetic testing not indicated’ is not an option. Which testing option would you select for this patient now?

| Whole genome sequencing |  |
| --- | --- |
| Whole exome sequencing |  |
| Cardiac panel test |  |

**QUESTION 5**

Consider the following hypothetical situation.

*A 44 year old male patient presents with shortness of breath following minimal exertion. He has two children and several siblings who also have children. He has a family history of hypertrophic cardiomyopathy. An echocardiogram shows asymmetric septal hypertrophy (maximum 16mm), confirmed by MRI scan. His coronary arteries are normal.*

Three tests are available that might be able to provide genetic information that could assist with the management of this patient and his family. You can order one of these tests, or you can decide that genetic testing is not indicated for this patient.

Please review these four testing options.


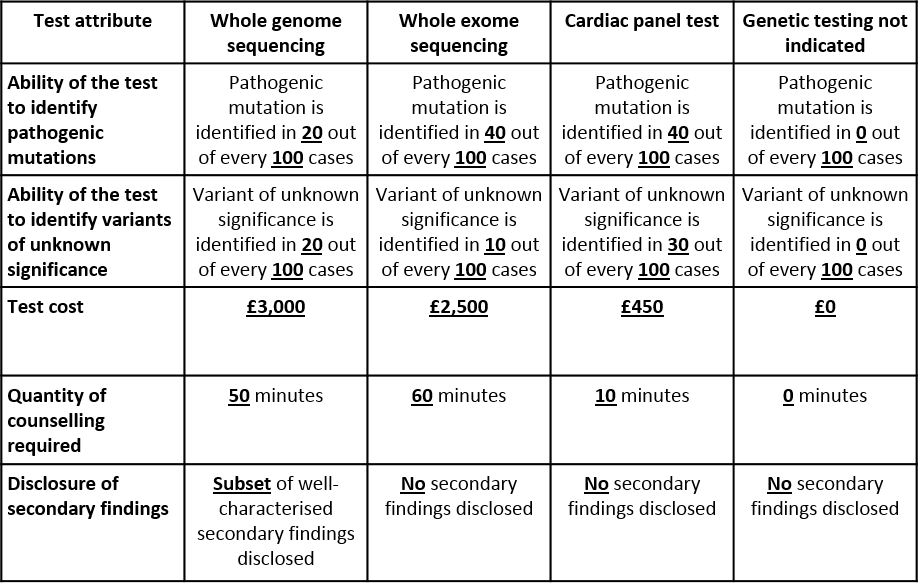


Which testing option would you select for this patient?

| Whole genome sequencing |  |
| --- | --- |
| Whole exome sequencing |  |
| Cardiac panel test |  |
| Genetic testing not indicated |  |

[If ‘Genetic testing not indicated’ is selected]

Now suppose that ‘Genetic testing not indicated’ is not an option. Which testing option would you select for this patient now?

| Whole genome sequencing |  |
| --- | --- |
| Whole exome sequencing |  |
| Cardiac panel test |  |

**QUESTION 6**

Consider the following hypothetical situation.

*A 44 year old male patient presents with shortness of breath following minimal exertion. He has two children and several siblings who also have children. He has a family history of hypertrophic cardiomyopathy. An echocardiogram shows asymmetric septal hypertrophy (maximum 16mm), confirmed by MRI scan. His coronary arteries are normal.*

Three tests are available that might be able to provide genetic information that could assist with the management of this patient and his family. You can order one of these tests, or you can decide that genetic testing is not indicated for this patient.

Please review these four testing options.


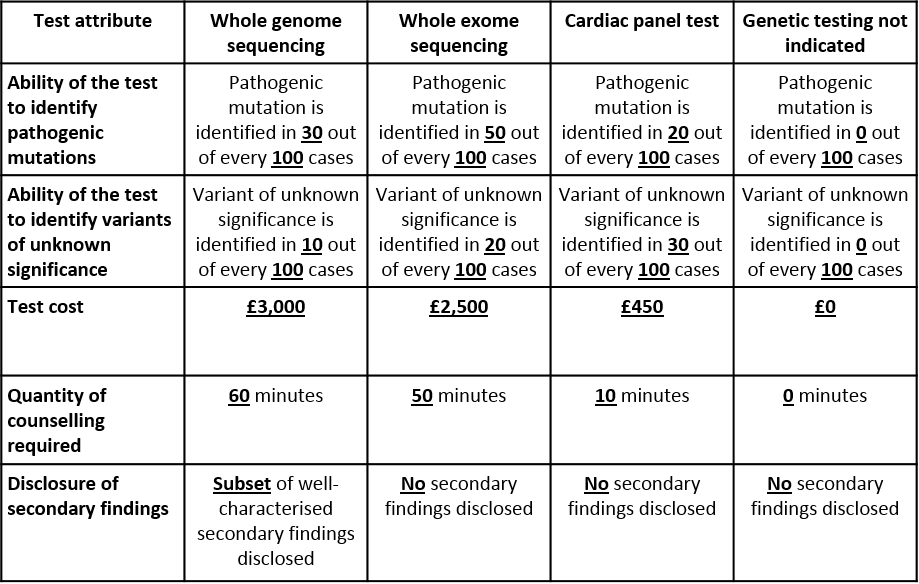


Which testing option would you select for this patient?

| Whole genome sequencing |  |
| --- | --- |
| Whole exome sequencing |  |
| Cardiac panel test |  |
| Genetic testing not indicated |  |

[If ‘Genetic testing not indicated’ is selected]

Now suppose that ‘Genetic testing not indicated’ is not an option. Which testing option would you select for this patient now?

| Whole genome sequencing |  |
| --- | --- |
| Whole exome sequencing |  |
| Cardiac panel test |  |

**QUESTION 7**

Consider the following hypothetical situation.

*A 44 year old male patient presents with shortness of breath following minimal exertion. He has two children and several siblings who also have children. He has a family history of hypertrophic cardiomyopathy. An echocardiogram shows asymmetric septal hypertrophy (maximum 16mm), confirmed by MRI scan. His coronary arteries are normal.*

Three tests are available that might be able to provide genetic information that could assist with the management of this patient and his family. You can order one of these tests, or you can decide that genetic testing is not indicated for this patient.

Please review these four testing options.


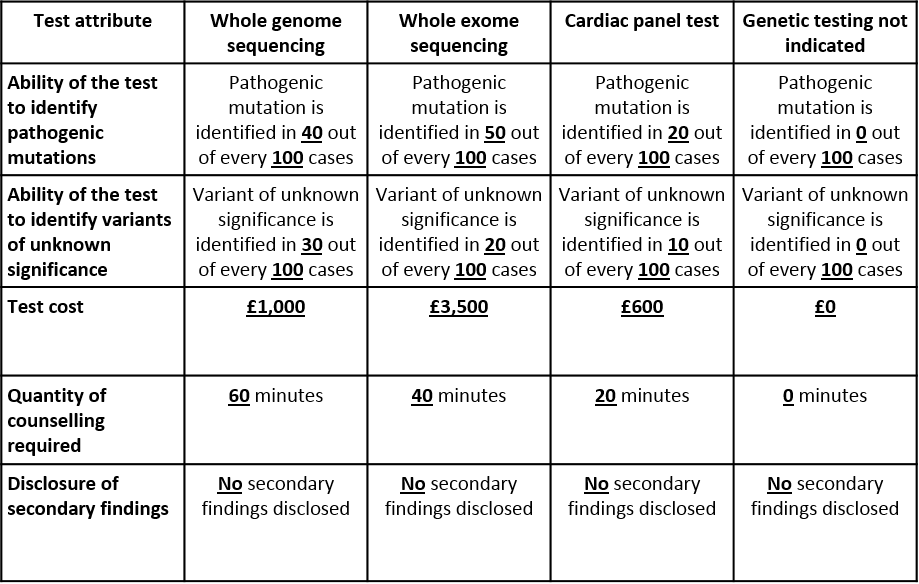


Which testing option would you select for this patient?

| Whole genome sequencing |  |
| --- | --- |
| Whole exome sequencing |  |
| Cardiac panel test |  |
| Genetic testing not indicated |  |

[If ‘Genetic testing not indicated’ is selected]

Now suppose that ‘Genetic testing not indicated’ is not an option. Which testing option would you select for this patient now?

| Whole genome sequencing |  |
| --- | --- |
| Whole exome sequencing |  |
| Cardiac panel test |  |

**QUESTION 8**

Consider the following hypothetical situation.

*A 44 year old male patient presents with shortness of breath following minimal exertion. He has two children and several siblings who also have children. He has a family history of hypertrophic cardiomyopathy. An echocardiogram shows asymmetric septal hypertrophy (maximum 16mm), confirmed by MRI scan. His coronary arteries are normal.*

Three tests are available that might be able to provide genetic information that could assist with the management of this patient and his family. You can order one of these tests, or you can decide that genetic testing is not indicated for this patient.

Please review these four testing options.


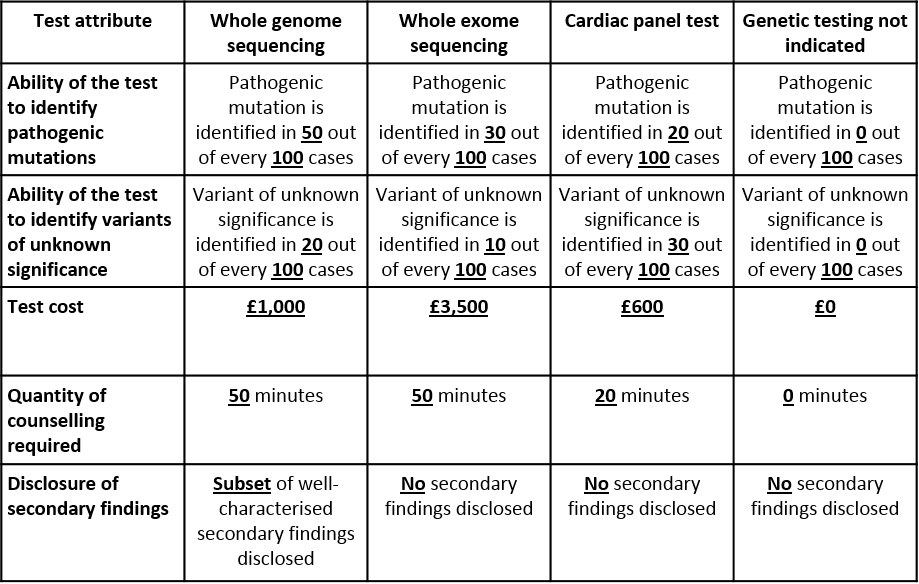


Which testing option would you select for this patient?

| Whole genome sequencing |  |
| --- | --- |
| Whole exome sequencing |  |
| Cardiac panel test |  |
| Genetic testing not indicated |  |

[If ‘Genetic testing not indicated’ is selected]

Now suppose that ‘Genetic testing not indicated’ is not an option. Which testing option would you select for this patient now?

| Whole genome sequencing |  |
| --- | --- |
| Whole exome sequencing |  |
| Cardiac panel test |  |

**QUESTION 9**

Consider the following hypothetical situation.

*A 44 year old male patient presents with shortness of breath following minimal exertion. He has two children and several siblings who also have children. He has a family history of hypertrophic cardiomyopathy. An echocardiogram shows asymmetric septal hypertrophy (maximum 16mm), confirmed by MRI scan. His coronary arteries are normal.*

Three tests are available that might be able to provide genetic information that could assist with the management of this patient and his family. You can order one of these tests, or you can decide that genetic testing is not indicated for this patient.

Please review these four testing options.


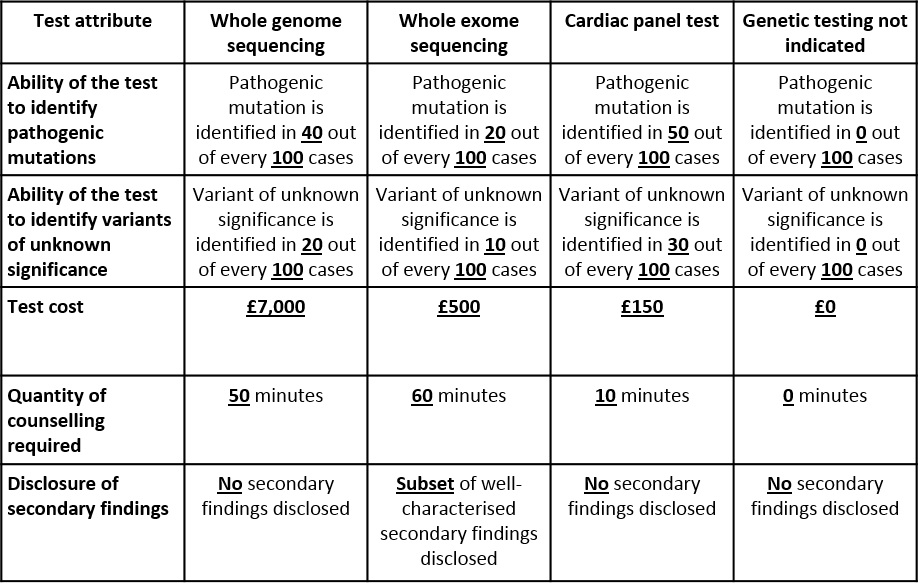


Which testing option would you select for this patient?

| Whole genome sequencing |  |
| --- | --- |
| Whole exome sequencing |  |
| Cardiac panel test |  |
| Genetic testing not indicated |  |

[If ‘Genetic testing not indicated’ is selected]

Now suppose that ‘Genetic testing not indicated’ is not an option. Which testing option would you select for this patient now?

| Whole genome sequencing |  |
| --- | --- |
| Whole exome sequencing |  |
| Cardiac panel test |  |

**QUESTION 10**

Consider the following hypothetical situation.

*A 44 year old male patient presents with shortness of breath following minimal exertion. He has two children and several siblings who also have children. He has a family history of hypertrophic cardiomyopathy. An echocardiogram shows asymmetric septal hypertrophy (maximum 16mm), confirmed by MRI scan. His coronary arteries are normal.*

Three tests are available that might be able to provide genetic information that could assist with the management of this patient and his family. You can order one of these tests, or you can decide that genetic testing is not indicated for this patient.

Please review these four testing options.


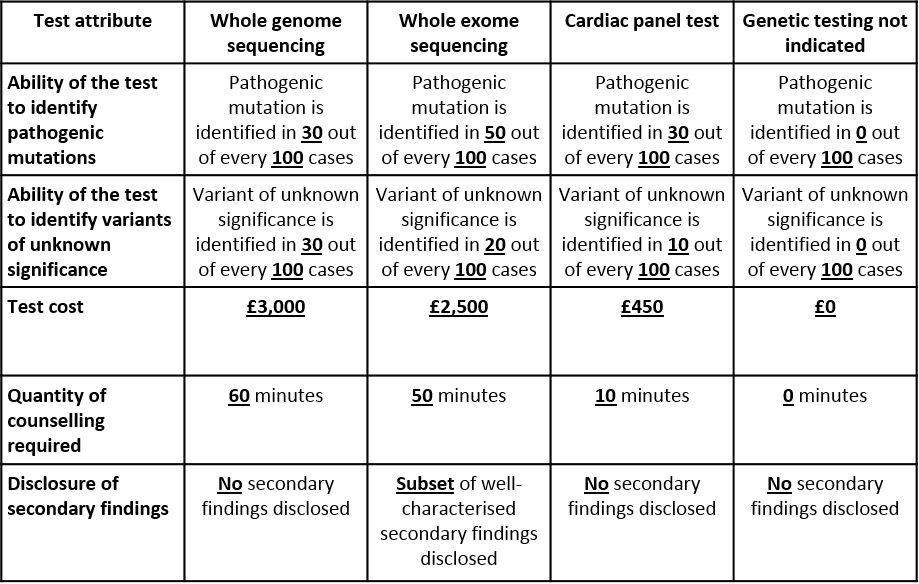


Which testing option would you select for this patient?

| Whole genome sequencing |  |
| --- | --- |
| Whole exome sequencing |  |
| Cardiac panel test |  |
| Genetic testing not indicated |  |

[If ‘Genetic testing not indicated’ is selected]

Now suppose that ‘Genetic testing not indicated’ is not an option. Which testing option would you select for this patient now?

| Whole genome sequencing |  |
| --- | --- |
| Whole exome sequencing |  |
| Cardiac panel test |  |

**QUESTION 11**

Consider the following hypothetical situation.

*A 44 year old male patient presents with shortness of breath following minimal exertion. He has two children and several siblings who also have children. He has a family history of hypertrophic cardiomyopathy. An echocardiogram shows asymmetric septal hypertrophy (maximum 16mm), confirmed by MRI scan. His coronary arteries are normal.*

Three tests are available that might be able to provide genetic information that could assist with the management of this patient and his family. You can order one of these tests, or you can decide that genetic testing is not indicated for this patient.

Please review these four testing options.


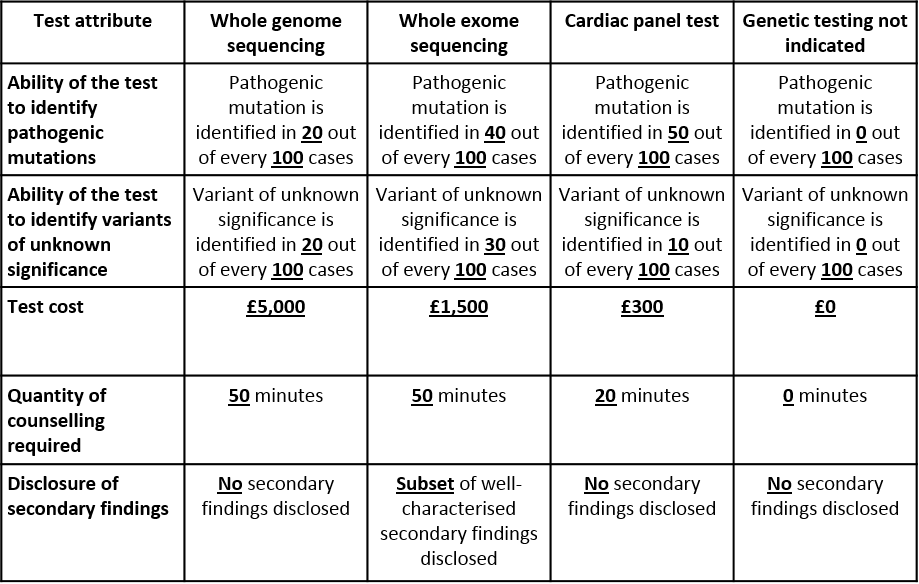


Which testing option would you select for this patient?

| Whole genome sequencing |  |
| --- | --- |
| Whole exome sequencing |  |
| Cardiac panel test |  |
| Genetic testing not indicated |  |

[If ‘Genetic testing not indicated’ is selected]

Now suppose that ‘Genetic testing not indicated’ is not an option. Which testing option would you select for this patient now?

| Whole genome sequencing |  |
| --- | --- |
| Whole exome sequencing |  |
| Cardiac panel test |  |

**QUESTION 12**

Consider the following hypothetical situation.

*A 44 year old male patient presents with shortness of breath following minimal exertion. He has two children and several siblings who also have children. He has a family history of hypertrophic cardiomyopathy. An echocardiogram shows asymmetric septal hypertrophy (maximum 16mm), confirmed by MRI scan. His coronary arteries are normal.*

Three tests are available that might be able to provide genetic information that could assist with the management of this patient and his family. You can order one of these tests, or you can decide that genetic testing is not indicated for this patient.

Please review these four testing options.


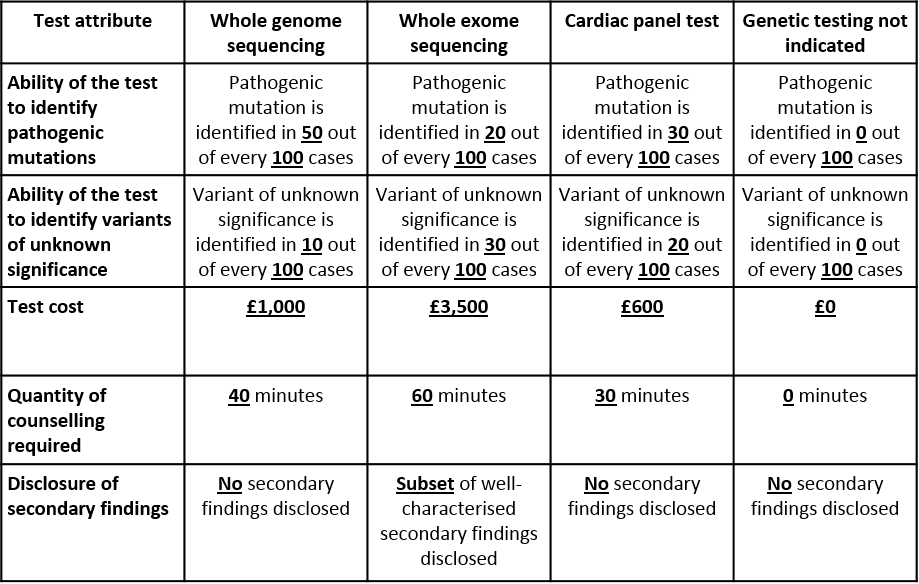


Which testing option would you select for this patient?

| Whole genome sequencing |  |
| --- | --- |
| Whole exome sequencing |  |
| Cardiac panel test |  |
| Genetic testing not indicated |  |

[If ‘Genetic testing not indicated’ is selected]

Now suppose that ‘Genetic testing not indicated’ is not an option. Which testing option would you select for this patient now?

| Whole genome sequencing |  |
| --- | --- |
| Whole exome sequencing |  |
| Cardiac panel test |  |

**ATTRIBUTE RANKING EXERCISE – PART TWO**

Thank you for completing the main part of the survey.

Now we would like you to complete the attribute ranking exercise again to see if your opinions have changed after answering these questions.

Please consider how important the five test attributes are to you and then rank them below.

Double-click or drag-and-drop items in the "Your choices" list to move them into the "Your ranking" list - your highest ranking (most important) item should be at the top of the list, moving through to your lowest ranking (least important) item at the bottom of the list.

[RANKING TABLE]

**RESPONDENT CHARACTERISTICS**

Now we would like to ask some questions about you.

All of the information that you provide will help us in our analysis, and all of your details will remain confidential.

If you do not wish to answer some of these questions you do not have to.

How many patients with suspected or confirmed inherited cardiovascular disease do you see in an outpatient or clinic setting in a typical month?

____ patients

Have you ever ordered a genetic or genomic test for a patient with an inherited cardiovascular disorder (suspected or confirmed)?

Yes

No

Don’t know

If yes, please describe the test(s) that you have ordered.

_____________

Have you ever ordered a genetic or genomic test for a patient with any other disorder (suspected or confirmed) i.e. not inherited cardiovascular disease?

Yes

No

Don’t know

If yes, please describe the test(s) that you have ordered.

_____________

Do you think genomic tests (e.g. whole genome sequencing, whole exome sequencing, cardiac panel tests) for patients with suspected or confirmed inherited cardiovascular disease should be made available in the UK NHS right now?

Yes

No

Uncertain

Please explain the reasoning behind your previous response.

If no, are there any scenarios (e.g. new evidence, change in test parameters) which would change your answer to the previous question to 'Yes'?

Yes

No

If yes, please describe these scenarios.

_____________

How old are you?

Under 25 years old

25-34 years old

35-44 years old

45-54 years old

55-64 years old

Over 64 years old

What is your gender?

Male

Female

Other

Prefer not to say

In which NHS Trust do you work?

___________________

Which of the following options best describes your main occupation?

Clinical geneticist

Genetic counsellor

Cardiologist

Other medical professional

Laboratory scientist

Academic researcher

Other

In what year did you qualify?

_________________

What is your primary clinical specialty? If you do not specialise in a particular clinical area, please answer ‘None’.

__________________

Please indicate how you first heard about this survey.

Via an email from the organisers of the Cardiovascular Disease Genomics England Clinical Interpretation Partnership (GeCIP)

Via the newsletter of the Association for Inherited Cardiac Conditions (AICC)

Other source – please describe

How easy or difficult did you find the questions in this survey?

[Likert scale 1-7, 1 = very easy, 7 = very difficult]

Are there any further comments that you would like to make regarding this survey?

________________

You have reached the end of the survey. Thank you for taking the time to participate. If you have any questions about this survey, please contact James Buchanan ([james.buchanan@dph.ox.ac.uk](mailto:james.buchanan@dph.ox.ac.uk)).

# Appendix 4: Dominance calculations and results

Methods

The choices that respondents made were reviewed to determine whether they exhibited dominant preferences (i.e. frequently selected the testing alternative with the best level of a particular attribute). Patterns of choice behaviour were specified for four of the five attributes and three of the four testing alternatives that would indicate dominant choice-making if observed for a respondent. As the expected impact on utility of a change in the ‘Level of disclosure of secondary findings’ attribute is unclear, a pattern of dominant choice behaviour was not specified for this attribute. These patterns were:

| **Respondent always selects the choice alternative…** | **Pattern of choice behaviour that would be observed if a respondent exhibited dominant preferences** | | | | | | | | | | | |
| --- | --- | --- | --- | --- | --- | --- | --- | --- | --- | --- | --- | --- |
|  | **Q1** | **Q2** | **Q3** | **Q4** | **Q5** | **Q6** | **Q7** | **Q8** | **Q9** | **Q10** | **Q11** | **Q12** |
| That identifies the most pathogenic mutations (PATHOGENIC) | P | P | G | X | X | E | E | G | P | E | P | G |
| That identifies the fewest variants of unknown significance (UNKNOWN) | N | N | N | N | N | N | N | N | N | N | N | N |
| That has the lowest cost (COST) | N | N | N | N | N | N | N | N | N | N | N | N |
| With the most counselling (COUNSEL) | E | G | G | E | E | G | G | X | E | G | X | E |
| That offers whole genome sequencing | G | G | G | G | G | G | G | G | G | G | G | G |
| That offers whole exome sequencing | E | E | E | E | E | E | E | E | E | E | E | E |
| That offers a cardiac panel test | P | P | P | P | P | P | P | P | P | P | P | P |
| Where no genetic test is indicated | N | N | N | N | N | N | N | N | N | N | N | N |

G = whole genome sequencing; E = whole exome sequencing; P = cardiac panel test; N = genetic testing not indicated; X = no dominant alternative for this choice.

Dominance scores were calculated for each respondent, by attribute or type of test. Respondents who had strictly dominant preferences for an attribute received a score of 12 (i.e. their choices exactly matched all 12 choices that a respondent with dominant preferences would make). For two attributes (PATHOGENIC and COUNSEL) there were 2 choices for which a dominant alternative could not be identified. The maximum dominance score for these attributes was 10. When a respondent made a choice that was not consistent with the existence of dominant preferences, their dominance score was reduced by 1.

Results

The table on the following page summarises the frequency of dominance scores for each attribute. No respondents were dominant for UNKNOWN, COST, COUNSEL, WGS, WES or NO TEST. There were 5 respondents who were dominant for PATHOGENIC and 4 respondents who were dominant for PANEL. The overall pattern of results suggests that the number of pathogenic mutations that are identified by a test and whether this test is a cardiac panel test are the most important factors for respondents deciding which test they would select. The other attributes and types of tests are of little importance when respondents make this choice decision.

| **Dominance score** | **Frequency ^a^** | | | | | | | |
| --- | --- | --- | --- | --- | --- | --- | --- | --- |
|  | **PATHOGENIC ^b^** | **UNKNOWN** | **COST** | **COUNSEL ^b^** | **WGS** | **WES** | **PANEL** | **NO TEST** |
| 0 | - | 33 | 33 | 15 | 5 | 13 | - | 33 |
| 1 | - | 2 | 2 | 18 | 2 | 6 | - | 2 |
| 2 | - | 2 | 2 | 4 | 10 | 4 | - | 2 |
| 3 | - | - | - | - | 20 | 9 | - | - |
| 4 | 5 | - | - | - | - | 4 | 1 | - |
| 5 | - | - | - | - | - | 1 | 4 | - |
| 6 | 11 | - | - | - | - | - | 3 | - |
| 7 | 4 | - | - | - | - | - | 9 | - |
| 8 | 7 | - | - | - | - | - | 8 | - |
| 9 | 5 | - | - | - | - | - | 4 | - |
| 10 | 5 | - | - | - | - | - | 3 | - |
| 11 |  | - | - |  | - | - | 1 | - |
| 12 |  | - | - |  | - | - | 4 | - |
| Mean score (SD) | 7.2  (1.9) | 0.2  (0.5) | 0.2  (0.5) | 0.7  (0.7) | 2.2  (1.1) | 1.7  (1.6) | 7.9  (2.1) | 0.2  (0.5) |

^a^ Number of respondents with a specific dominance score. ^b^ The maximum dominance score was 10, not 12. SD = standard deviation. The maximum dominance score is shaded for each attribute and testing alternative.

# Appendix 5: Development of the mixed logit model of choice behaviour

Mixed logit regression analysis was used to construct our model of choice behaviour. Mixed logit regression analysis allows researchers to account for preference heterogeneity amongst respondents by permitting model parameters to be randomly distributed. They key consequence of this approach is that a mixed logit regression returns both mean coefficients for all the alternative-specific constants and attributes in a DCE, and also standard deviations for these coefficients, which account for preference heterogeneity.

Mixed logit regression analysis was operationalised using the mixlogit user written command in Stata (StataCorp. 2015. Stata Statistical Software: Release 14. College Station, TX: StataCorp LP) (1). This command requires users to follow three steps:

1. Select the appropriate number of Halton draws for the estimation process;
2. Determine which parameters will be fixed and which will be random;
3. Adjust the regression model to allow for correlation between attributes.

Step 1

Our initial regression used 50 Halton draws, treated all parameters as random and did not adjust for correlation between attributes. We then increased the number of Halton draws in 50 draw increments up to 500 draws, then in 100 draw increments up to 1,000 draws. The model that provided the best fit, as measured by the Akaike and Bayesian Information Criterion, was the model that used 500 draws.

Step 2

In Step 2, we relaxed the assumption that all parameters were random. We considered as random those parameters that were associated with standard deviations that were significant (P≤5%). All other parameters were treated as fixed parameters. In the final regression, five parameters were considered to be random (the alternative-specific constants for whole genome sequencing and the panel test, PATHOGENIC, UNKNOWN and COST). The remaining three attributes (the alternative-specific constant for whole exome sequencing, COUNSEL and SECONDARY) were considered to be fixed.

Step 3

In Step 3, we adjusted our model to allow for correlation between attributes.

# Appendix 6: Attribute rankings, before and after completing the choice questions

The table below describes the results of the ranking exercise. Prior to completing the choice questions, the attribute that was ranked by respondents as being the most important was the ability of the test to identify pathogenic mutations, with the least important attribute being disclosure of secondary findings. After completing the choice questions, there was little change in the mean importance scores; the only difference was that respondents placed slightly more importance on the ability of the test to identify variants of unknown significance (VUS), and slightly less importance on the quantity of counselling received.

| **Attribute** | **Before completing choice questions** | | **After completing choice questions** | |
| --- | --- | --- | --- | --- |
|  | **Mean score (SD)** | **Ranking** | **Mean score (SD)** | **Ranking** |
| Ability of the test to identify pathogenic mutations | 1.0 (0.0) | 1 | 1.0 (0.2) | 1 |
| Test cost | 2.7 (0.9) | 2 | 2.5 (0.9) | 2 |
| Quantity of counselling received | 3.4 (1.0) | 3 | 3.5 (0.9) | 4 |
| Ability of the test to identify variants of unknown significance | 3.6 (1.1) | 4 | 3.4 (1.1) | 3 |
| Disclosure of secondary findings | 4.4 (0.8) | 5 | 4.5 (0.7) | 5 |

SD = standard deviation

# Appendix 7: Further information on respondent use of genetic and genomic tests, and on their preferences regarding the future use of genomic testing

Respondents were given the opportunity to provide further comments in response to three questions in the survey. These responses are listed below, by respondent ID. Negative responses are indicated with red text.

**Question 1**: Have you ever ordered a genetic or genomic test for a patient with an inherited cardiovascular disorder (suspected or confirmed)? If yes, please describe the test(s) that you have ordered.

| **ID** | **Response to question** | **Comment** |
| --- | --- | --- |
| 17 | Yes | WGS and gene panel testing |
| 18 | Yes | Point mutation tests, single gene tests, panels, MLPA, array CGH, clinical exomes |
| 20 | Yes | HCM, Brugada Syndrome, Inherited long-QT syndrome, LMNA cardiomyopathy |
| 22 | Yes | Cardiac panel-familial hypercholesterolaemia (LDLR; apoB; PCSK9; LDLRAP-1), cardiac panel-lipoprotein lipase (LPL; ApoC2; ApoA5; LMF-1; GPIHBP1), WGS |
| 24 | Yes | Cardiac panel |
| 25 | Yes | Single gene or gene panel tests for LQT, HCM, DCM, Brugada, ARVC |
| 26 | Yes | Mostly testing for inherited arrhythmia syndromes (ARVC, CPVT, LQTS, Brugada syndrome) |
| 30 | Yes | HCM panel, occasional LQT |
| 31 | Yes | Cardiac panel, WGS, cascade screening in families |
| 33 | Yes | Gene panels |
| 34 | Yes | Aortopathy panel |
| 36 | Yes | Cardiac panels for HCM, LQT, aortopathy, ARVC, single gene tests for Brugada, DCM |
| 37 | Yes | Individual genes (Sanger), gene panels of varying sizes, research WGS |
| 39 | Yes | Panel tests for cardiomyopathies & inherited arrhythmias, predictive tests for the same disorders |
| 42 | Yes | Cardiac panel |
| 43 | Yes | Cardiac gene panel disease specific |
| 47 | Yes | Research via 100,000 genomes project and DDD study, NHS panel tests for aortopathy, Marfan via single gene analysis |
| 50 | Yes | 100,000 Genomes Project recruitment, panel tests |
| 51 | Yes | Cardiac panel tests, single gene tests (historically), recruitment to 100,000 Genomes Project |
| 54 | No | - |
| 55 | Yes | Routinely order cardiac panel test (aortic subset), also enrol patients in 100,000 Genomes Project routinely for aortic and congenital heart disease sub-domains |
| 57 | Yes | Cardiac panel tests |
| 58 | Yes | Panel |
| 59 | No | - |
| 60 | Yes | Familial Hypercholesterolaemia |
| 62 | Yes | Mainly panel tests, some WGS or WES for unexplained paediatric CM |
| 63 | Yes | Panel testing for all cardiac inherited conditions |
| 65 | Yes | Panel testing |
| 67 | Yes | Specific gene testing, panel testing, predictive testing, mitochondrial testing |
| 70 | Yes | Single gene, panel, exome, genome even array CGH |
| 71 | Yes | Panel & exomes via BRRD, or genomes via GEL |
| 72 | Yes | Panel tests, medical exome, WGS (research) |
| 74 | Yes | Panel, WES, WGS, array CGH |
| 78 | Yes | Cardiac panel |
| 82 | Yes | Custom panel, exome panel, WGS panel (100,000 Genomes Project) |
| 83 | Yes | Cardiac panel |
| 87 | Yes | Panels, single gene tests in the past |

**Question 2**: Have you ever ordered a genetic or genomic test for a patient with any other disorder (suspected or confirmed) i.e. not inherited cardiovascular disease? If yes, please describe the test(s) that you have ordered.

| **ID** | **Response to question** | **Comment** |
| --- | --- | --- |
| 17 | Yes | Chromosome analysis e.g. Down syndrome or FISH for Chromosome 22q11 microdeletion |
| 18 | Yes | Chromosome breakage, FISH, karyotype, methylation, uPD |
| 20 | No | - |
| 22 | Yes | Genetic testing for severe obesity (e.g. leptin; MCR4) or lipodystrophy (lamin A/C; PPAR-gamma) |
| 24 | No | - |
| 25 | No | - |
| 26 | Yes | Barth syndrome testing, 22q deletion syndrome testing, Fibrillin gene |
| 30 | No | - |
| 31 | No | - |
| 33 | Yes | Single gene tests, gene panels, virtual gene panels through an exome, WES, WGS |
| 34 | Yes | Microarray, breast/ovarian cancer panel, Lynch syndrome panel, renal tumours panel, phaeochromocytoma/paraganglioma panel |
| 36 | Yes | Single gene tests, panels, WES, WGS for many different disorders |
| 37 | Yes | Genes, panels, microarrays, karyotype, biochemical |
| 39 | Yes | Far too many to list |
| 42 | No | - |
| 43 | Yes | Genetic testing for many inherited conditions |
| 47 | Yes | Many - range of tests as appropriate to working in Clinical Genetics |
| 50 | Yes | Am clinical geneticist, so lots! |
| 51 | Yes | Many (am clinical geneticist) |
| 54 | Yes | Pharmacogenetics, primarily for warfarin metabolism |
| 55 | No | - |
| 57 | Yes | Adult onset dominant conditions like cancer and HD. Carrier tests for CF and Sickle cell. Testing for known familial conditions like Fragile X, DMD etc |
| 58 | No | - |
| 59 | No | - |
| 60 | No | - |
| 62 | No | - |
| 63 | No | - |
| 65 | No | - |
| 67 | No | - |
| 70 | No | - |
| 71 | No | - |
| 72 | Yes | Occasional specific gene tests or panel tests |
| 74 | Yes | See previous response - many panels |
| 78 | No | - |
| 82 | No | - |
| 83 | Yes | Panels and WES |
| 87 | Yes | Huge range from single gene to WGS |

**Question 3**: Do you think genomic tests (e.g. whole genome sequencing, whole exome sequencing, cardiac panel tests) for patients with suspected or confirmed inherited cardiovascular disease should be made available in the UK NHS right now? Please explain the reasoning behind your response.

| **ID** | **Response to question** | **Comment** |
| --- | --- | --- |
| 17 | Yes | Yes but I think there should be a minimum training undergone by any clinician ordering such a test. |
| 18 | Yes | Standard of care |
| 20 | Yes | When screening for common mutations is negative, more detailed testing is sometimes useful with appropriate genetic expertise / advice |
| 22 | Yes | NICE FH guidance (CG71) (cost-effective to do comprehensive genetic testing) already published in 2009 but widely ignored in England as opposed to Scotland, Wales, or Northern Ireland. Good data for HOCM, channelopathies as well. |
| 24 | Yes | Very important for individual risk profiling and cascade screening |
| 25 | Yes | Cardiac panel tests are available |
| 26 | Yes | Given the patients I manage, to have the test available would make my job easier. I do believe that the tests should not be available to all practitioners, but only to practitioners with experience managing patients with inherited cardiac conditions. |
| 30 | Yes | It helps cascade screening greatly - especially in large families |
| 31 | Yes | To use as second-line when a cardiac panel has been undertaken and no pathogenic variants were found |
| 33 | Yes | Cardiac panel tests are already available in the UK and are extremely useful in confirming familial risk, and thus for providing family cascade screening. They are less useful in the diagnosis of the proband, although they can confirm whether an ICC is a genetic one, or possibly non-genetic, or secondary to another disorder. |
| 34 | Yes | Yes, but should be offered to patients and families with a clear phenotype. The tests should be mainstreamed and the cost of interpreting variants of unknown significance should be factored in. |
| 36 | Yes | For diagnostic & predictive testing purposes. Some genes also provide additional phenotypic information that is actionable e.g. LAMIN |
| 37 | Yes | Multiple genes causing same phenotype, clinically easy to miss phenocopies e.g. Fabry, good signal to noise |
| 39 | Yes | Finding a genetic diagnosis in an affected individual allows for tailored screening of family members, so those at risk continue with screening and be managed appropriately e.g. with beta blockers, and those not at risk can stop being screened. Also allows for reproductive decision-making in some families, especially those with tragic histories. |
| 42 | Yes | - |
| 43 | Yes | These tests may provide information that is important to patient management and predictive tests for family members |
| 47 | Yes | For those individuals with a clearly pathogenic variant, the ability to rule family members into or out of screening is appropriate. |
| 50 | Yes | Is already available. Complex output needs interpretation. Despite rhetoric of mainstreaming many cardiologists do not feel equipped to interpret many of these reports, so need cardiogeneticists. Invention of X-ray led to lots of mainstream specialities being able to diagnose clear fractures, but still need radiologists to interpret more complex or subtle abnormalities. Same in genomics. |
| 51 | Yes | - |
| 54 | Yes | They are useful in some circumstances. |
| 55 | Yes | I find it enormously helpful to have a confirmed diagnosis with aortopathy patients as it guides medical and surgical management and allows for cascade screening. I would favour in the long term whole genome sequencing once staff have appropriate training in counselling and interpretation |
| 57 | Yes | Testing is important for clarifying risk and tailoring screening which allows more cost effective provision of services. This is important for the adjustment of individuals to the condition and adherence to screening and lifestyle recommendations. Testing also allows to identify those at risk who may have been unaware thus reducing the incidence of SCD. |
| 58 | Yes | - |
| 59 | Yes | I refer patients to genetics team who order tests. Testing is extremely helpful in the management of families with suspected ICC. |
| 60 | Yes | For FH the tests are clearly highly cost effective. |
| 62 | Yes | Panel tests are the most cost-effective way of obtaining a molecular diagnosis. WES/WGS are almost certainly cost-effective in rare undiagnosed diseases (including syndromic and paediatric cardiomyopathy). |
| 63 | Yes | Speed up referral process and allow ICC specialists to direct access tests |
| 65 | Yes | It is important for management of the ICC, family screening and risk assessment. |
| 67 | No | Complexity of interpretation of results and ethical issues surrounding disclosure of secondary information |
| 70 | Yes | It is helping large numbers of relatives of patients with ICC get personalised medical advice and care. It is informing the management of smaller numbers of patients with conditions where there is an important genotype-phenotype correlation. |
| 71 | Yes | Because they are available |
| 72 | Yes | Shown to be clinically effective and cost effective in selected settings |
| 74 | Yes | - |
| 78 | Yes | More cost effective overall |
| 82 | Yes | Used carefully, taking into account the issues you have highlighted, they offer advantages over serial single gene tests. The ability to test more genes in a single test leads to greater number of diagnoses, more quickly than was the case years ago. BUT I am not convinced that cardiologists generally have the skills and knowledge to know how to use these tests. |
| 83 | Yes | - |
| 87 | No | Too expensive. Panel is far more cost-effective and as good. Panel has better coverage of the relevant genes, costs less and is quicker to interpret |

# Appendix 8: Distributions of individual-level coefficients

Density function – individual-level coefficients for the alternative-specific constant for WGS

Density function – individual-level coefficients for the alternative-specific constant for panel testing

Density function – individual-level coefficients for the ‘Ability of the test to identify pathogenic mutations’ attribute

Density function – individual-level coefficients for the ‘Ability of the test to identify variants of unknown significance’ attribute

Density function – individual-level coefficients for the ‘Test cost’ attribute

# Appendix 9: Observed and predicted uptake probabilities

| **Ch** | **Test** | **Test attributes and levels** | | | | | **Utility score** | | **% of respondents who chose test** | | **Probability of uptake** | |
| --- | --- | --- | --- | --- | --- | --- | --- | --- | --- | --- | --- | --- |
|  |  | **Pathogenic mutation identified in X out of every 100 cases** | **VUS identified in X out of every 100 cases** | **Cost** | **Counselling** | **Secondary findings disclosed** | **Mean** | **SD** | **Mean** | **SD** | **Mean** | **SD** |
| 1 | WGS | 30 | 30 | £5,000 | 40 mins | None | -29.2 | 33.8 | 0.0% | 0.0% | 0.0% | 0.0% |
|  | WES | 30 | 20 | £1,500 | 50 mins | Subset | 9.5 | 9.6 | 2.7% | 16.4% | 1.4% | 8.3% |
|  | Panel | 40 | 10 | £300 | 30 mins | None | 35.1 | 16.2 | 97.2% | 16.4% | 98.4% | 9.6% |
|  | None | 0 | 0 | £0 | 0 mins | None | -0.1 | 0.0 | 0.0% | 0.0% | 0.2% | 1.3% |
| 2 | WGS | 40 | 30 | £7,000 | 60 mins | Subset | -41.4 | 47.3 | 0.0% | 0.0% | 0.0% | 0.0% |
|  | WES | 20 | 10 | £500 | 40 mins | None | 13.3 | 6.6 | 2.7% | 16.4% | 1.6% | 8.5% |
|  | Panel | 50 | 20 | £150 | 30 mins | None | 43.5 | 21.2 | 97.2% | 16.4% | 98.4% | 8.7% |
|  | None | 0 | 0 | £0 | 0 mins | None | -0.1 | 0.0 | 0.0% | 0.0% | 0.0% | 0.1% |
| 3 | WGS | 50 | 10 | £7,000 | 50 mins | Subset | -26.0 | 44.0 | 16.2% | 37.4% | 16.3% | 36.7% |
|  | WES | 30 | 30 | £500 | 40 mins | None | 17.7 | 10.0 | 8.1% | 27.7% | 5.5% | 19.8% |
|  | Panel | 30 | 20 | £150 | 30 mins | None | 24.7 | 11.7 | 73.0% | 45.0% | 77.5% | 39.6% |
|  | None | 0 | 0 | £0 | 0 mins | None | -0.1 | 0.0 | 2.7% | 16.4% | 0.7% | 3.7% |
| 4 | WGS | 20 | 10 | £5,000 | 40 mins | None | -33.6 | 35.5 | 0.0% | 0.0% | 0.0% | 0.0% |
|  | WES | 40 | 30 | £1,500 | 60 mins | None | 15.3 | 12.0 | 0.0% | 0.0% | 1.7% | 9.1% |
|  | Panel | 40 | 20 | £300 | 20 mins | None | 33.6 | 15.5 | 100.0% | 0.0% | 98.2% | 9.6% |
|  | None | 0 | 0 | £0 | 0 mins | None | -0.1 | 0.0 | 0.0% | 0.0% | 0.1% | 0.6% |
| 5 | WGS | 20 | 20 | £3,000 | 50 mins | Subset | -17.3 | 19.9 | 0.0% | 0.0% | 0.0% | 0.0% |
|  | WES | 40 | 10 | £2,500 | 60 mins | None | 10.5 | 14.7 | 13.5% | 34.7% | 12.7% | 32.1% |
|  | Panel | 40 | 30 | £450 | 10 mins | None | 30.6 | 14.0 | 81.1% | 39.7% | 87.1% | 32.3% |
|  | None | 0 | 0 | £0 | 0 mins | None | -0.1 | 0.0 | 5.4% | 22.9% | 0.2% | 1.0% |
| 6 | WGS | 30 | 10 | £3,000 | 60 mins | Subset | -6.3 | 17.6 | 0.0% | 0.0% | 0.0% | 0.0% |
|  | WES | 50 | 20 | £2,500 | 50 mins | None | 18.4 | 16.2 | 64.9% | 48.4% | 65.2% | 47.2% |
|  | Panel | 20 | 30 | £450 | 10 mins | None | 11.7 | 5.8 | 32.4% | 47.5% | 34.4% | 47.4% |
|  | None | 0 | 0 | £0 | 0 mins | None | -0.1 | 0.0 | 2.7% | 16.4% | 0.4% | 1.7% |
| 7 | WGS | 40 | 30 | £1,000 | 60 mins | None | 17.7 | 12.5 | 37.8% | 49.2% | 37.1% | 47.7% |
|  | WES | 50 | 20 | £3,500 | 40 mins | None | 9.5 | 20.1 | 32.4% | 47.5% | 35.0% | 46.2% |
|  | Panel | 20 | 10 | £600 | 20 mins | None | 14.3 | 5.6 | 27.0% | 45.0% | 27.3% | 43.3% |
|  | None | 0 | 0 | £0 | 0 mins | None | -0.1 | 0.0 | 2.7% | 16.4% | 0.6% | 3.2% |
| 8 | WGS | 50 | 20 | £1,000 | 50 mins | Subset | 30.7 | 17.5 | 81.1% | 39.7% | 85.1% | 34.5% |
|  | WES | 30 | 10 | £3,500 | 50 mins | None | -7.8 | 21.3 | 2.7% | 16.4% | 0.8% | 4.4% |
|  | Panel | 20 | 30 | £600 | 20 mins | None | 9.3 | 5.4 | 13.5% | 34.7% | 13.8% | 34.4% |
|  | None | 0 | 0 | £0 | 0 mins | None | -0.1 | 0.0 | 2.7% | 16.4% | 0.3% | 1.7% |
| 9 | WGS | 40 | 20 | £7,000 | 50 mins | None | -38.0 | 46.8 | 0.0% | 0.0% | 0.0% | 0.0% |
|  | WES | 20 | 10 | £500 | 60 mins | Subset | 11.5 | 6.6 | 0.0% | 0.0% | 0.2% | 0.7% |
|  | Panel | 50 | 30 | £150 | 10 mins | None | 42.9 | 20.5 | 100.0% | 0.0% | 99.8% | 1.0% |
|  | None | 0 | 0 | £0 | 0 mins | None | -0.1 | 0.0 | 0.0% | 0.0% | 0.0% | 0.3% |
| 10 | WGS | 30 | 30 | £3,000 | 60 mins | None | -11.4 | 18.3 | 0.0% | 0.0% | 0.0% | 0.0% |
|  | WES | 50 | 20 | £2,500 | 50 mins | Subset | 18.5 | 16.2 | 40.5% | 49.8% | 40.6% | 47.9% |
|  | Panel | 30 | 10 | £450 | 10 mins | None | 26.2 | 10.7 | 59.5% | 49.8% | 59.2% | 48.0% |
|  | None | 0 | 0 | £0 | 0 mins | None | -0.1 | 0.0 | 0.0% | 0.0% | 0.2% | 1.2% |
| 11 | WGS | 20 | 20 | £5,000 | 50 mins | None | -37.1 | 36.1 | 0.0% | 0.0% | 0.0% | 0.0% |
|  | WES | 40 | 30 | £1,500 | 50 mins | Subset | 16.4 | 12.0 | 0.0% | 0.0% | 0.8% | 5.0% |
|  | Panel | 50 | 10 | £300 | 20 mins | None | 45.5 | 21.0 | 100.0% | 0.0% | 99.2% | 5.1% |
|  | None | 0 | 0 | £0 | 0 mins | None | -0.1 | 0.0 | 0.0% | 0.0% | 0.0% | 0.1% |
| 12 | WGS | 50 | 10 | £1,000 | 40 mins | None | 34.1 | 18.3 | 86.5% | 34.7% | 85.2% | 34.5% |
|  | WES | 20 | 30 | £3,500 | 60 mins | Subset | -23.1 | 24.6 | 0.0% | 0.0% | 0.0% | 0.0% |
|  | Panel | 30 | 20 | £600 | 30 mins | None | 20.3 | 9.3 | 13.5% | 34.7% | 14.8% | 34.5% |
|  | None | 0 | 0 | £0 | 0 mins | None | -0.1 | 0.0 | 0.0% | 0.0% | 0.0% | 0.0% |

Ch = Choice. Mins = Minutes. Panel = Panel test. SD = Standard deviation. VUS = Variants of unknown significance. WES = Whole exome sequencing. WGS = Whole genome sequencing. Tests with the highest probability of uptake in each choice are highlighted.

Commentary

The table on the previous page reports the estimated utility associated with the alternatives in each choice question and the observed rate of uptake for each alternative, then uses the utility estimates to calculate the probability of uptake for each alternative.

Of the 12 choices that were made by each respondent, panel testing had the highest observed uptake in 8 choices, WGS had the highest observed uptake in 3 choices and WES had the highest observed uptake in the remaining choice.

Observed uptake rates for specific choices were very closely aligned with predicted uptake rates. Predicted uptake rates ranged from 0% to 85% for WGS, 0% to 65% for WES, 14% to 100% for panel testing and 0% to 1% for no test. For WGS, the test with the highest predicted uptake (choice 12) identifies pathogenic mutations in 50 out of 100 cases, identifies VUS in 10 out of 100 cases, costs £1,000, is supported by 40 minutes of counselling and doesn’t disclose any secondary findings. For WES, the test with the highest predicted uptake (choice 6) identifies pathogenic mutations in 50 out of 100 cases, identifies VUS in 20 out of 100 cases, costs £2,500, is supported by 50 minutes of counselling and doesn’t disclose any secondary findings. For panel testing, the test with the highest predicted uptake (choice 9) identifies pathogenic mutations in 50 out of 100 cases, identifies VUS in 30 out of 100 cases, costs £150, is supported by 10 minutes of counselling and doesn’t disclose any secondary findings.

To understand the likely uptake of these tests in current practice, levels were set for each test that reflected current knowledge of test attributes. These levels are defined in the following table.

| **Test** | **Test attributes and levels** | | | | | **Utility score** | | **Probability of uptake** | |
| --- | --- | --- | --- | --- | --- | --- | --- | --- | --- |
|  | **Pathogenic mutation identified in X out of every 100 cases** | **VUS identified in X out of every 100 cases** | **Cost** | **Counselling (mins)** | **Secondary findings disclosed** | **Mean** | **SD** | **Mean** | **SD** |
| WGS | 50 | 55 | £5,000 | 60 | Subset | -18.5 | 30.8 | 0.1% | 0.6% |
| WES | 45 | 35 | £2,500 | 60 | Subset | 9.0 | 15.2 | 1.4% | 4.1% |
| Panel | 40 | 15 | £300 | 30 | None | 33.9 | 15.9 | 98.1% | 6.2% |
| None | 0 | 0 | £0 | 0 | None | -0.1 | 0.0 | 0.4% | 2.3% |

Panel = Panel test. SD = Standard deviation. VUS = Variants of unknown significance. WES = Whole exome sequencing. WGS = Whole genome sequencing

We made the following assumptions when defining these levels:

- The number of cases in which pathogenic mutations are identified was set to be 50 out of every 100 for WGS, 45 out of every 100 for WES and 40 out of every 100 for panel testing.
- The number of cases in which VUS are identified was set to be 55 out of every 100 for WGS, 35 out of every 100 for WES and 15 out of every 100 for panel testing.
- WGS costs £5,000 (lower than current estimates in laboratories at the University of Oxford but higher than the predicted cost at which WGS will likely be rolled out in clinical practice in the UK NHS). The cost of WES is set to be half this amount (£2,500), with the cost of panel testing approximately equal to current estimates in laboratories at the University of Oxford.
- The maximum amount of counselling is provided for each test.
- A subset of well-characterised secondary findings is disclosed for WGS and WES.

These assumptions were combined with the coefficients generated in our regression analysis for all attributes – including the alternative specific constants – to calculate the utility associated with each testing alternative for each survey respondent. We then used these utility estimates to predict the likely uptake of each option. Panel testing had the highest mean utility score (33.9), then WES (9.0), then no testing (-0.1), then WGS (-18.5). The large standard deviations for the utility scores for WGS and WES highlight the heterogeneity in respondent preferences. For WGS, the maximum utility score was 26.3 and the minimum was -73.2, while for WES, the maximum and minimum were 29.5 and -14.8, respectively. Given these test specifications, panel testing would be the preferred test of all 37 respondents and has the highest predicted uptake rate (98%).

# Appendix 10: Extending the model to explore the drivers of response heterogeneity

Given the heterogeneity that was observed both in the choices made by respondents and in the model of choice behaviour that was developed based on these choices, we extended our base case model to explore the potential drivers of this heterogeneity. Our process was as follows:

1. We identified the respondent characteristics that we considered to be the most likely drivers of respondent heterogeneity. Three characteristics were selected:
   1. The number of patients with suspected or confirmed inherited cardiovascular disease that respondents saw in a typical month;
   2. Respondent gender, and
   3. Respondent year of qualification.
2. These characteristics were interacted with the five variables with coefficients that suggested preference heterogeneity, thus generating five interaction terms for each characteristic. These variables were:
   1. The alternative-specific constant for WGS;
   2. The alternative-specific constant for panel testing;
   3. The ‘Ability of the test to identify pathogenic mutations’ attribute;
   4. The ‘Ability of the test to identify variants of unknown significance’ attribute, and
   5. The ‘Test cost’ attribute.
3. The five interaction terms for each characteristic were each introduced to the base case model separately as new random parameters. Seven interaction terms with significant standard deviations were retained:
   1. The number of patients with suspected or confirmed inherited cardiovascular disease that respondents saw in a typical month AND
      1. The alternative-specific constant for panel testing;
      2. The ‘Ability of the test to identify variants of unknown significance’ attribute;
      3. The ‘Test cost’ attribute;
   2. Gender AND
      1. The alternative-specific constant for panel testing;
   3. Respondent year of qualification AND
      1. The alternative-specific constant for WGS;
      2. The alternative-specific constant for panel testing, and
      3. The ‘Ability of the test to identify pathogenic mutations’ attribute.
4. The interaction terms that were retained following step 3 were then all included as random parameters in the base case model. Although we explored several combinations of some or all of these attributes, we were unable to achieve convergence with this model, likely due to the sample size available to conduct this analysis.

# References

1. Hole AR. Fitting mixed logit models by using maximum simulated likelihood. Stata Journal. 2007;7(3):388-401.
